# Efficacy of volatile pyrethroid spatial repellents to reduce exposure to mosquito and sand fly vectors and human infection risk: a systematic review and meta-analysis

**DOI:** 10.1186/s13071-026-07393-2

**Published:** 2026-06-18

**Authors:** Ngwa Niba Rawlings, Mark Stephen Bailey, Orin Courtenay

**Affiliations:** 1 Department of Environmental Health, Defence Medical Services, Ministry of Defence, Coventry, UK; 2 School of Life Sciences, University of Warwick, Coventry, UK; 3 Warwick Medical School, University of Warwick, Coventry, UK; 4 Academic Department of Military Medicine, Royal Centre for Defence Medicine, Birmingham, UK; 5 Zeeman Institute, University of Warwick, Coventry, UK

**Keywords:** Spatial repellents, Volatile pyrethroid, Transfluthrin, Metofluthrin, Vector control, Malaria, Arboviruses, Leishmaniasis, Protective efficacy, Mosquitoes, Sand flies

## Abstract

**Background:**

Volatile pyrethroid spatial repellents (VPSRs) have emerged as promising complementary tools for vector control. This systematic review and meta-analysis evaluated the protective efficacy (PE) of VPSRs against mosquito- and sand-fly-borne human infections, and against vector numbers in traps.

**Methods:**

Clinical infection and entomological studies that evaluated VPSRs against human infection with *Plasmodium*, arboviruses (Dengue and Zika), and *Leishmania*, and against their respective vectors, were systematically reviewed. Random-effects meta-analyses estimated pooled PE values and explored moderators including vector trapping method, deployment setting (indoor/outdoor), and active ingredient (a.i.) concentration.

**Results:**

A total of 49 VPSR studies containing 154 datasets met the inclusion criteria, comprising 6 trials (7 datasets) against human infection incidence, and 43 entomological studies mostly of mosquitoes (147 datasets); 73.5% tested active ingredients transfluthrin and 22.4% tested metofluthrin.

Pooled estimates from transfluthrin VPSR trials indicated protective effects of 50% (95% CI 21–69%) against *Plasmodium* (*n* = 3) and 34% (95% CI 10–52%) against arboviruses (Dengue and Zika, *n* = 1). Evidence for *Leishmania* infection incidence was limited to a single trial (PE = 48%, 95% CI 26–63%). Metofluthrin showed no effect against *Plasmodium* (*n* = 1).

Exposure to VPSRs was associated with substantial reductions in trapped mosquito and sand fly numbers. The comparative protection inferred from the entomological data was variably dependent on trap type: the mosquito data gave pooled mean PE values of 57–62% by human landing catches; 35–55% using Centers for Disease Control light traps (CDC-LT); and 47–88% using CDC-LT co-located with sources of CO^2^. The wider range of pyrethroid compounds tested against *Phlebotomus* sand flies (*n* = 4) gave trap-specific PE values of 93%, 53%, and 78%, respectively. VPSR deployment in outdoor settings appeared not to be generally inferior to indoor deployment, and efficacy values tended not to differ significantly between mosquito genera. The relationship between the level of repellency (and/or lethality) and VPSR a.i. concentration is not clear, though higher concentrations (≥ 20% w/v transfluthrin or metofluthrin) appeared to be less effective.

**Conclusions:**

The collective results demonstrate that transfluthrin-based VPSRs were associated with reduced vector numbers and reduced *Plasmodium* infection incidence, with likely effectiveness also against arboviral and *Leishmania* infection incidence. However, existing evidence for reduced infection incidence is very limited despite strong entomological effects. More studies to evidence-base and strengthen conclusions are needed on many key aspects of VPSR products for programmatic and sustainable scale-up.

**Supplementary Information:**

The online version contains supplementary material available at https://doi.org/10.1186/s13071-026-07393-2.

## Background

Vector-borne diseases spread by arthropod vectors account for 17% of all infectious diseases of humans and are responsible for 700,000 human deaths per year [1]. Among these, mosquito and Phlebotomine sand fly species transmit devastating etiological agents causing human diseases including malaria, leishmaniasis, arboviruses, and phleboviruses [1, 2]. Community health vector control programmes conventionally deploy indoor residual spraying (IRS) or outdoor ultra-low-volume spatial spraying (ULV) of insecticides, insecticide-treated bednets (ITNs), or long-lasting insecticide-treated bednets (LLINs). These interventions have achieved significant reductions in human-vector exposure or/and subclinical and clinical infections [3–6]. However, as IRS, ITNS, and LLINs predominantly target indoor (endophilic/endophagic) nocturnal vectors, they have limited impact on more exophilic/exophagic vectors that blood-feed outside, such as zoophilic blood-feeders [7, 8]. Thus, an outstanding challenge is to expand the toolbox to combat vectors and transmission that occurs exterior to residential structures.

One recognized complementary vector control approach is to deploy volatile pyrethroid spatial repellents (VPSRs). VPSRs are esters that in a vapor state create a chemical aerosol atmosphere that interferes with host-seeking behavior, inhibits blood-feeding, induces behavioral repellency from the chemical source, and in some cases, enhances vector knockdown and 24-h mortality. Pyrethroid insecticides bind to and disrupt insect voltage-gated sodium channels in nerve cell membranes causing prolonged sodium influx, repetitive nerve firing, and hyperexcitation. Unlike IRS, ITNS, and LLINs, VPSRs do not require vectors to have physical contact with treated surfaces but rather act at a distance. Active ingredients (a.i.) include metofluthrin, transfluthrin, allethrin, prallethrin, and meperfluthrin, however, the majority of published studies of VPSRs report on the efficacy of transfluthrin or metofluthrin.

In 2025, the World Health Organization (WHO) announced recommendation and prequalification listings for two VPSR emanator products containing transfluthrin, SC Johnson Guardian™, and SC Johnson Mosquito Shield™ (S. C. Johnson & Son, Inc., Wisconsin, USA), for protection against malaria [3]. On the basis of the collective “moderate certainty evidence” the recommendation is conditional, limited to their deployment indoors only, and as a supplementary intervention only in locations already receiving wide-coverage with IRS/ITNs/LLINs [3]. The effectiveness of VPSRs against human infection and disease, and against more exophilic vectors, remains to be more widely evidenced for unconditional recommendation by the WHO [3].

A recent systematic review and meta-analysis published in eBioMedicine synthesized the entomological evidence on volatile pyrethroid spatial repellents (VPSRs), focusing primarily on their effects on mosquitoes on the basis of studies published up to September 2023 [9]. That review reported consistent reductions in mosquito biting rates across multiple vector genera, although with substantial variability between studies. The present review complements and extends that work by integrating epidemiological endpoints (infection incidence and transmission metrics where reported) alongside entomological outcomes, and by including available evidence against *Leishmania* infection incidence and phlebotomine sand fly vectors, updating the literature to August 2025.

Currently, estimates of the protective efficacy (PE) of VPSRs are generated largely from entomological data rather than on the basis of human clinical/infection trials per se, as availability of the latter are limited [10–14], and entomological studies are generally quicker, logistically easier, and cheaper to perform [15–29]. Vector enumeration using different trapping methods, e.g., human landing catches, light-traps, baited light-traps, and sticky traps, is not independent of the biological processes governing a trap’s “attraction” to vectors or probability of capture. Consequently, trap counts vary in their epidemiological interpretation and in their hierarchical strength of evidence to infer a VPSR protective effect against human infection. It is therefore prudent to scrutinize comparatively the PE values according to the entomological method(s) underlying vector counts. Higher-order entomological indicators such as sporozoite rates and entomological inoculation rates (EIR) provide stronger epidemiological linkage to infection but are rarely incorporated into entomological VPSR studies to date.

By consolidating recently available data, this systematic review and meta-analysis examines the protective effects of VPSRs on human infection risk and entomological markers, differentiating the range of trapping methods and VPSR deployment settings (inside versus outside) while also assessing heterogeneity associated with VPSR delivery devices and insecticide a.i. concentrations. This review also includes the limited literature on VPSRs tested against leishmania infection incidence and attributed changes in phlebotomine sand fly numbers.

## Methods

The review was registered on International Prospective Register of Systematic Reviews (PROSPERO; CRD420251010630), and followed the Preferred Reporting Items for Systematic Review and Meta-Analysis (PRISMA) guidelines [30, 31] (Table S1).

### Inclusion and exclusion criteria

The inclusion criteria included randomized controlled trials (RCTs), cluster randomized controlled trials (cRCTs), and other nonrandomized controlled trials (nRCTs) that evaluated the effects of VPSRs on mosquito- or sand-fly-borne human infection risk compared with appropriate controls, and attributed changes in mosquito and sand fly numbers captured in traps. Studies published between January 2000 and August 2025, irrespective of language (non-English papers were translated) or publication status, were examined against the inclusion criteria. VPSR studies that were not appropriately controlled or lacked epidemiological or entomological outcome measures of interest were excluded.

### Definitions

For the purposes of the analyses, the environmental setting where the VPSRs were tested were classified as “indoor” or “outdoor” where indoor settings are spaces within physical structures such as houses, tents, enclosed rooms, or other habitations, where the repellent acts within a fully or semi-enclosed environment. This category included the reported “semi-field” studies using experimental huts or shelters enclosed with biosecure mosquito-proof netting into which known numbers of wild-caught or colony-reared mosquitoes were released. Outdoor settings are spaces not confined by physical structures and where environmental factors such as airflow (potentially increasing volatile insecticide dispersal and dilution) or wild vector access (entry and exit) are not prevented. This category comprised the reported “field studies” on verandas, in courtyards, and in peridomestic open settings.

The entomological trap types were distinguished as human landing catches (HLC), in which trained collectors captured mosquitoes landing on exposed parts of their body before the vectors bite; CDC light traps (CDC-LT), which utilize light and suction to capture insects; and CDC light traps enhanced with CO_2_ (dry ice or CO_2_ dispenser, *n* = 2), or natural odor cues from humans sleeping in close vicinity to the CDC-LT (*n* = 4) (CDC-LT + CO_2_). Two additional techniques to capture sand flies were reported: manual aspiration to collect resting or host-seeking sand flies that alight on surfaces and suction traps involving a continuous fan-driven airflow to passively capture flying sand flies.

### Data sources and extraction

Medical Literature Analysis and Retrieval System Online (MEDLINE) (Ovid), Embase (Ovid), Web of Science, and LILACS (BIREME) were searched between January 2000 to August 2025, using terms “randomised controlled trial”, “cluster randomised controlled trial”, ‘”intervention “, “experiment”, “spatial repellent”, “volatile pyrethroid”, “metofluthrin”, “transfluthrin”, “mosquito*”, “anopheles”, “culex”, “aedes”, “sand fl*”, “phlebotomine”, “vector control”, “malaria”, “vaporizer mat*”, and “emanator” (see Supplementary Information for full search strategy). Two clinical trial registries, the WHO International Clinical Trials Registry Platform (ICTRP) and ClinicalTrials.gov, as well as preprint servers (bioRxiv and medRxiv), article reference lists, and Google Scholar Citations were manually searched up to 30 August 2025.

Two reviewers (N.R. and O.C.) screened the titles and abstracts, and then the full text of potentially eligible studies, to complete a standardized inclusion criteria form. Extracted data included experimental design details (unit of analysis, randomization method, clustering adjustments for cRCTs, sample size, study duration, recruitment methods, blinding procedures, and ethical approval), participant characteristics (location, demographics, inclusion/exclusion criteria, subgroups), and intervention details (environmental location, description of treatment and control groups, treatment duration, and compliance). Entomological trap type, a.i. concentration, VPSR deployment setting, dispenser type, and insect taxa were also recorded.

### Effect measures

Published entomological and epidemiological effect measures included incidence rate ratio (IRR), hazard ratio (HR), and odds ratio (OR), along with 95% confidence intervals (CI), and any adjustments for clustering, intraclass correlation coefficient (ICC), or coefficient of variation (COV). If time-dependent data were reported, person-time at risk or rate ratios (RR) with variance measures were also extracted. The control groups were reported to receive placebo repellents or no intervention.

### Data analysis

Meta-analyses were performed by fitting random effects models (REMs) to the entomological or epidemiological measures to calculate the mean (and 95% CIs) protective efficacy (PE) of VPSRs on human infection risk, and PEs based on changes in mosquito/sand fly numbers in traps. As studies reported different effect measures, ORs and HRs were converted to RRs following [32], and transformed to log RR and corresponding standard error (SE) values for the meta-analyses. The resulting pooled *log*RR value was then back-transformed and effect estimate tested using the *z*-statistic. The PE values, reported here as percentages, were calculated as PE = ([1 − RR] × 100%). PEs generated from individual studies/datasets with pooled results are illustrated as Forest plots.

All analyses were performed using STATA v.18 software (StataCorp LP, College Station, TX, USA).

#### Heterogeneity

We anticipated heterogeneity due to differences in vector species, intervention delivery formats, insecticide concentration, and sampling methods. Between-study consistency was assessed using four heterogeneity statistics shown beneath each pooled estimate (*I*^2^, *τ*^2^, *H*^2^, and Cochran’s *Q*). Heterogeneity was considered minimal when *τ*^2^ was near 0, *I*^2^ was close to 0%, H^2^ was close to 1, and *Q* was nonsignificant (*P* > 0.05), and substantial when *τ*^2^ was larger, *I*^2^ exceeded 75%, H^2^ was > 1, and *Q* was significant (*P* < 0.05) (see forest plots) [33, 34]. A *Q* statistic is also reported within each subgroup to quantify heterogeneity among studies in that subgroup, alongside an overall test of group differences indicating whether subgroup pooled effects differ. Post-estimation pairwise comparisons of PE estimates between categories were performed using lincom routines in STATA.

#### Subgroup and sensitivity analysis

Subgroup analysis was conducted to further explore sources of heterogeneity in PE values associated with the product a.i. concentration, vector trapping method, VPSR diffuser type, deployment setting, and vector taxon. Exploration of additional study-level covariates such as geography and study year, though available for all studies, were not considered meaningful explanatory variables of between-study variation in effect estimates. Insecticide resistance status of vector populations was not analyzed because it was not reported sufficiently consistently across studies to support reliable modeling within outcomes.

For meta-analyses including at least ten datasets, “leave-one-out” sensitivity analyses were conducted by sequentially excluding each study in turn to examine for potential changes in the summary effect estimates and confidence intervals.

### Quality assessment

#### Risk of bias

Risk of bias was assessed using the National Institutes of Health (NIH) Quality Assessment Tool [42] to ensure a consistent appraisal across the largely trap-based entomological intervention studies, often clustered by site. Risk of bias (RoB) 2/Risk Of Bias In Non-Randomized Studies - of Interventions (ROBINS-I) were not used because most domain questions are tailored to participant-level trial processes and were not consistently reported or directly applicable to trap-based entomological field designs. A series of signaling questions informed an overall study quality rating of good, fair, or poor, considering key methodological aspects; these ratings are not additive, but each domain contributes to the overall judgment (Table S2).

#### Reporting bias

For meta-analyses with ≥ 10 studies, publication bias was also assessed by visual inspection of funnel plots of effect estimates against standard errors (shown in Additional file 1 Figs. S9–S12), and by the *P*-values of the Egger’s test statistic [35].

## Results

**Fig. 1:**
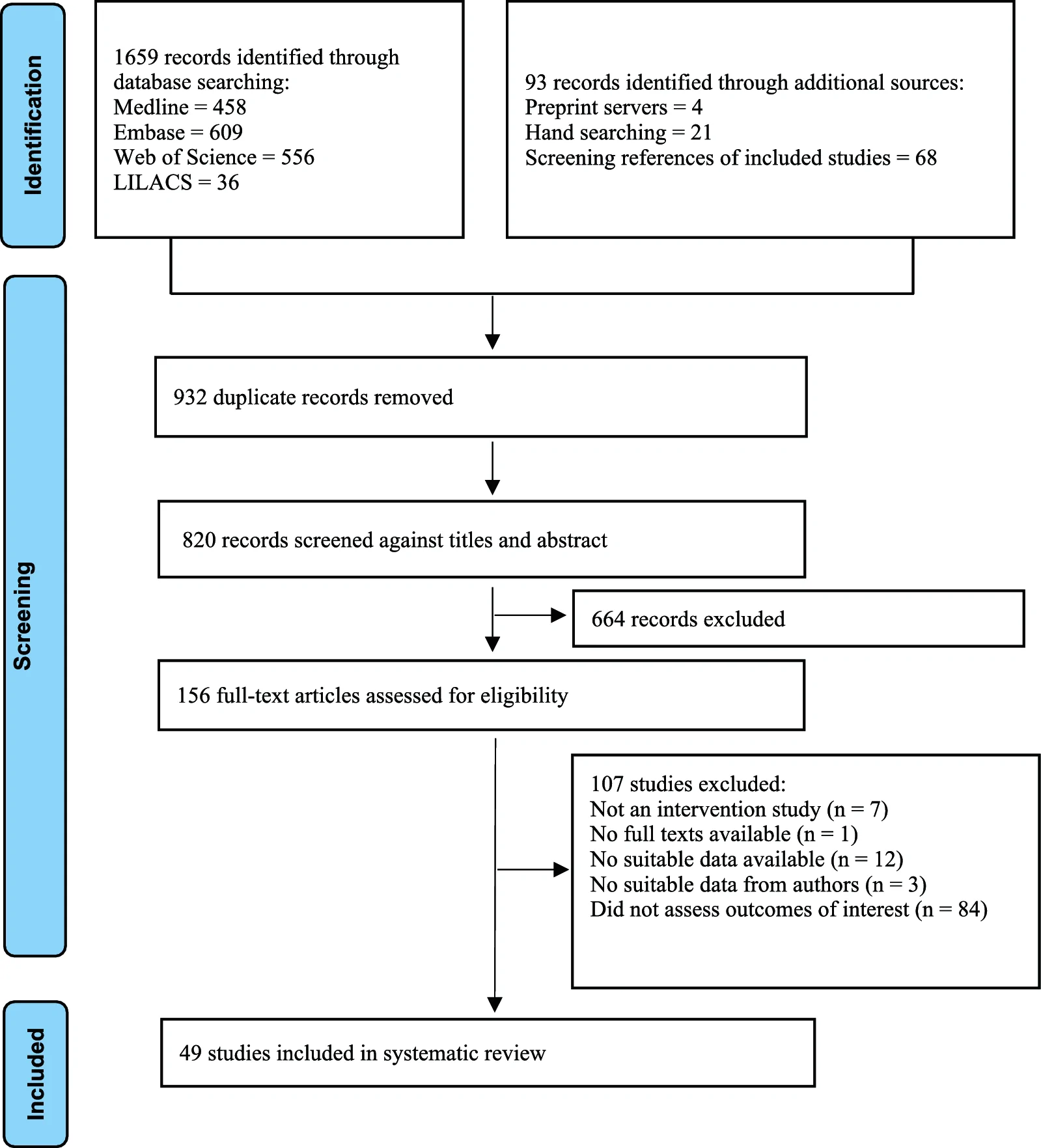
PRISMA flowchart showing process of study inclusion and exclusion

A total of 1752 studies were identified through the literature search. After removing duplicates, 820 studies were screened by title and abstract. Of these, 664 were excluded for being unrelated to the focus of the review. The full text of 156 studies was reviewed for eligibility, and 49 studies met the inclusion criteria (Fig. 1).

### Description of included studies

The 49 eligible studies contained 154 datasets available for analyses. Most studies tested transfluthrin (*n* = 36, 73.5%), followed by metofluthrin (*n* = 11, 22.4%), and one study each tested prallethrin (2%) and cis–trans allethrin (2%). Of these, 44 studies (89.8%) investigated effects on mosquito vectors, while 5 studies (10.2%) focused on sand fly vectors.

A subset of six studies assessed VPSR effects on human infection incidence (40–12,360 households per study): four studies investigated first-time *Plasmodium* infection incidence, confirmed by microscopy and rapid diagnostic tests [11–14], one study examined *Aedes*-borne viral infections causing dengue or Zika (ABV) [10], and one study evaluated *Leishmania* infection incidence causing cutaneous leishmaniasis, confirmed by parasite presence in skin scrapings [36]. Two of the four *Plasmodium* studies reported sporozoite rates and estimated entomological inoculation rates (EIR) [12, 13]. Of the five mosquito-related infection studies, four were cRCTs [10–13] and one was a RCT [14]; these were conducted in Southeast Asia (*n* = 3: one in China, two in Indonesia), sub-Saharan Africa (Kenya, *n* = 1), and South America (Peru, *n* = 1) (Table S3). Most tested transfluthrin (*n* = 4), while the other tested metofluthrin (*n* = 1). The single study on sand flies was a cRCT conducted in northeast Syria (Asia) and tested transfluthrin delivered by passive diffuser (Mosquito Shield™) [36].

The 49 entomological studies identified trap types; most common were HLCs (*n* = 37, 75.5%), followed by CDC-LT + CO_2_ (*n* = 6, 12.2%), CDC-LT (*n* = 5, 10.2%), and manual aspirators/suction traps (*n* = 1, 2%). Studies targeted one or more mosquito genera (*Anopheles, Aedes, Culex, Mansonia*) and sand fly genera (*Phlebotomus*). VPSR devices were deployed in indoor (*n* = 19, 38.8%), outdoor (*n* = 20, 40.8%), or tested concurrently in indoor and outdoor (*n* = 10, 20.4%) settings as already defined in the same study.

The most frequently reported VPSR a.i. concentrations were categorized as ≤ 5% w/v (transfluthrin *n* = 29, 59.2%; metofluthrin *n* = 4, 8.2%; prallethrin *n* = 1, 2%); > 5 to ≤ 10% w/v (transfluthrin *n* = 2, 4.1%; metofluthrin *n* = 3, 6.1%); > 10 to ≤ 20% w/v (transfluthrin *n* = 4, 8.2%); and > 20% w/v (transfluthrin *n* = 2, 4.1%; metofluthrin *n* = 3, 6.1% and cis–trans allethrin *n* = 1, 2%). One study [37] did not report the concentration (Table S3).

VPSR diffusers varied in design; passive diffusers were the most common (*n* = 38, 77.6%), followed by coils (*n* = 6, 12.2%), Thermacell emanators (*n* = 3, 6.1%), and aerosol spray (*n* = 1, 2%). Passive diffuser materials were mainly fabric (hessian, sisal, cotton, or nylon), followed by polyethylene; resin and paper were used rarely (Table S3).

In terms of study design, 27 studies (55.1%) were RCTs [14, 15, 38–62], 5 (10.2%) were cRCTs [10–13, 36], and 17 (34.7%) were nRCTs [16, 37, 63–78]. The majority of studies were conducted in Africa (*n* = 28, 57.1%), including Tanzania (*n* = 19) [15, 38, 39, 43–47, 50, 52, 55–59, 68, 69, 73, 74], Kenya (*n* = 4) [11, 49, 65, 71], Zambia (*n* = 2) [37, 53], Benin (*n* = 2) [42, 51], and Zimbabwe (*n* = 1) [70]. Asia accounted for 13 studies (26.5%): 5 in Thailand [16, 60, 66, 72, 75], 2 each in Cambodia [61, 67], Indonesia [12, 13], and Israel [77, 78], and 1 each in Syria [36] and China [14]. Four were in the Americas (8.2%): two in Peru [10, 41], one each in the USA [63], Mexico [40], and Haiti [54], and three (6.1%) in Europe: one each in Spain [48], Greece [76], and Turkey [62]. In Australia, there was one (2%) [64].

### Meta-analyses

#### Effect of VPSRs on mosquito-borne human infection incidence

**Fig. 2:**
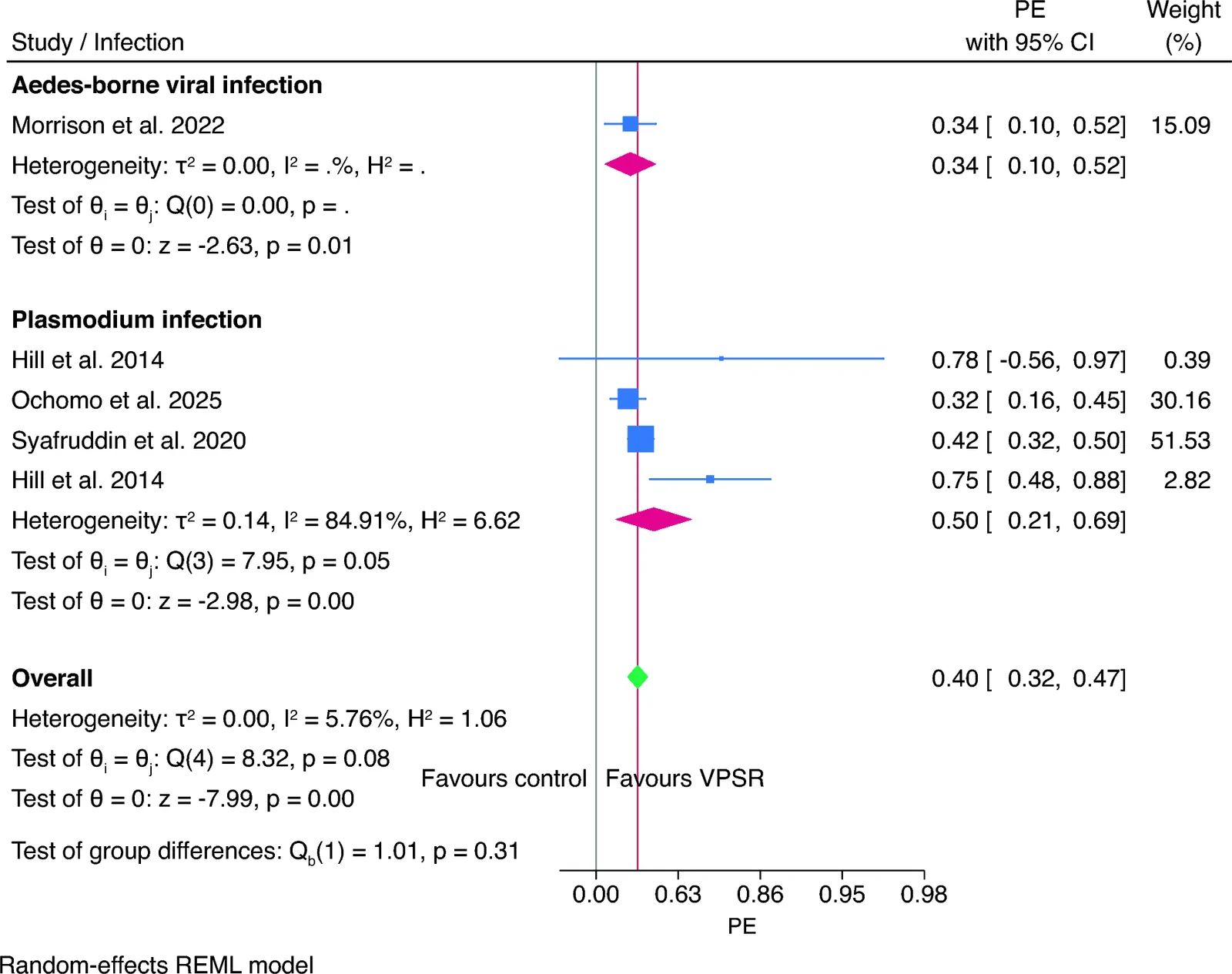
Forest plot showing the efficacy of transfluthrin VPSRs on infection incidence of *Plasmodium* and *Aedes*-borne infections (dengue and Zika viruses). Studies appearing more than once indicate multiple independent datasets reported within the same study. The size of the blue squares (mean estimates) reflects the sample size. *VPSR* volatile pyrethroid spatial repellent, *PE* protective efficacy

Meta-analysis of four datasets from three studies evaluating transfluthrin VPSRs on *Plasmodium* infection incidence resulted in a pooled PE of 50% (95% CI 21–69%, *P* < 0.001; Fig. 2); not dissimilar to PE = 34% (95% CI 10−52%, *P* = 0.01) against ABV infection incidence in the single transfluthrin study (Fig. 2). The single metofluthrin VPSR trial [12] reported a statistically nonsignificant PE of 35% (95% CI −3.75% to 91%, *P* = 0.67) against *Plasmodium*.

#### Effect of transfluthrin on mosquito numbers according to trapping method

**Fig. 3:**
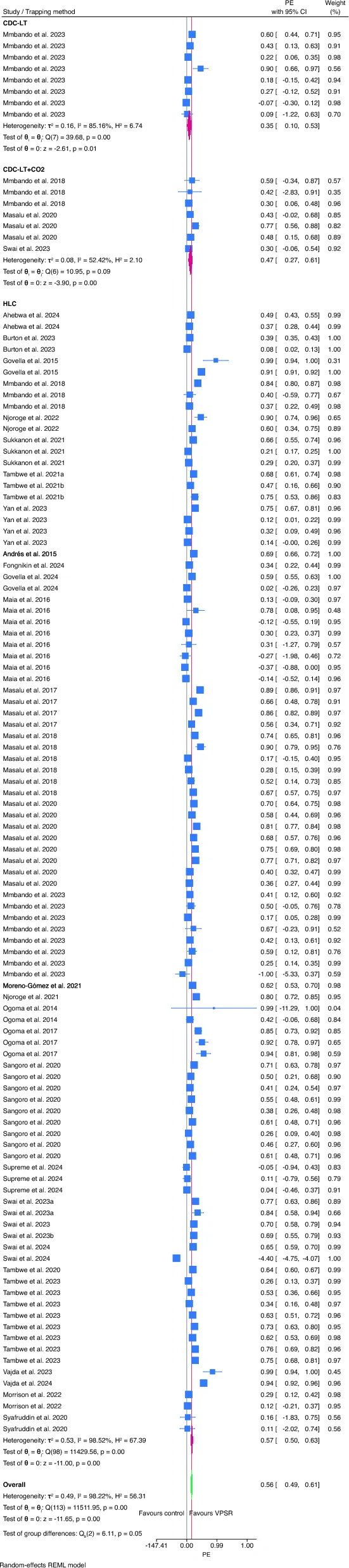
Forest plot showing the efficacy of transfluthrin on mosquito numbers according to the entomological trapping method. Studies appearing more than once indicate multiple independent datasets reported within the same study. The size of the blue squares (mean estimates) reflects the sample size. *HLC* human landing catch, *CDC-LT* Centers for Disease Control light trap, *CDC-LT + CO₂* Centers for Disease Control carbon dioxide–baited light trap, *VPSR* volatile pyrethroid spatial repellent, *PE* protective efficacy

Reported absolute counts of mosquito numbers were measured by HLC (99 datasets, 32 studies), CDC-LT (8 datasets, 1 study), and CDC-LT + CO_2_ (7 datasets, 3 studies). PE estimates generated from each trapping method indicated significant protection, but their values varied slightly (*Qβ*(2) = 6.11, *P* = 0.05) (Fig. 3).

Meta-regression and post-estimation contrasts provided no evidence that trapping method moderated PE estimates (Wald *χ*^2^(2) = 1.93, *P* = 0.381; *r*^2^ = 0.19%), confirmed by pairwise analyses (*P* > 0.191 in each case).

**Fig. 4:**
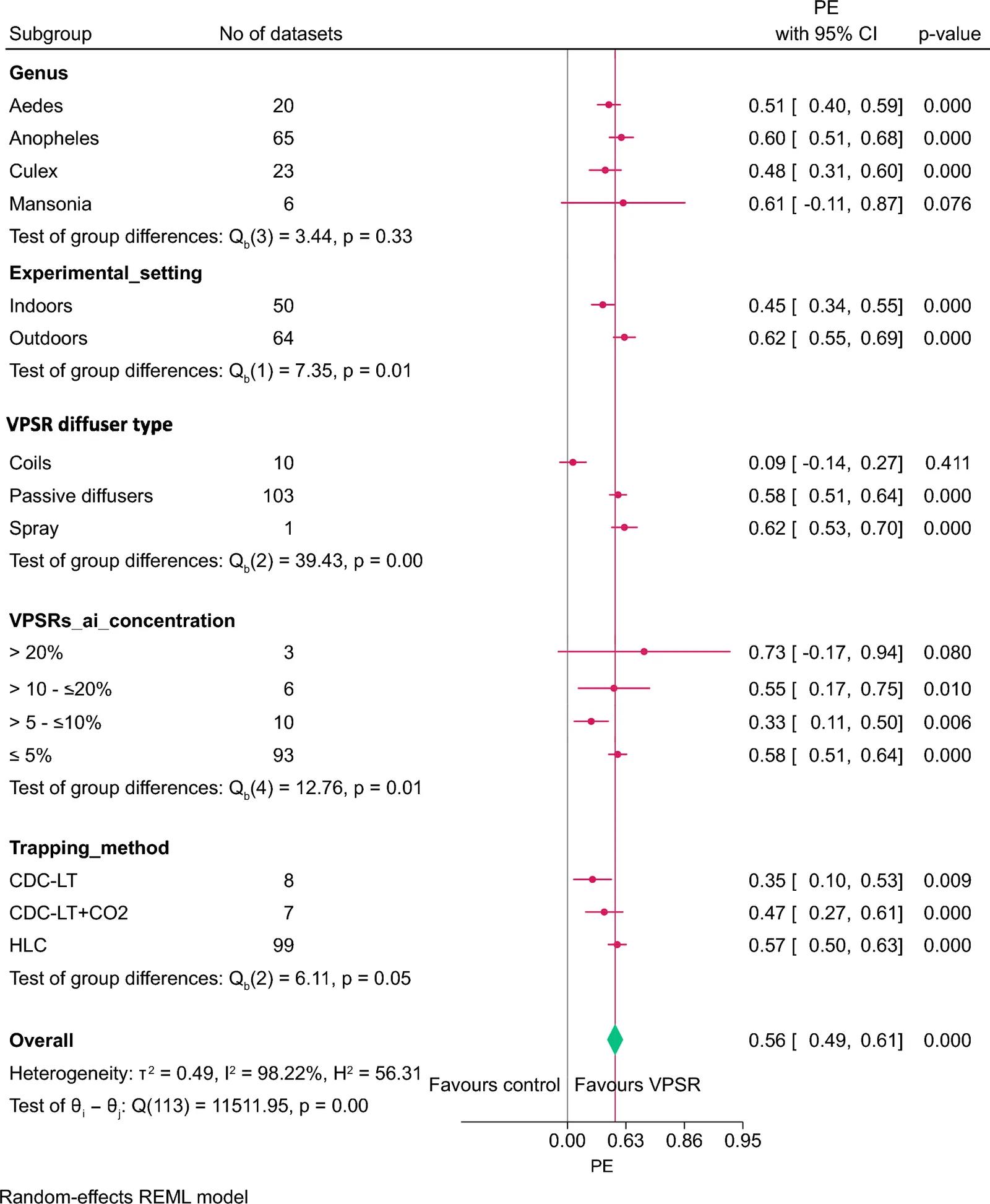
Analysis of the efficacy of transfluthrin VPSRs against mosquito numbers according to variable subgroups. *HLC* human landing catch, *CDC-LT* Centers for Disease Control light trap, *CDC-LT + CO₂* Centers for Disease Control carbon dioxide–baited light trap, *a.i.* active ingredient (w/v), *VPSR* volatile pyrethroid spatial repellent, *PE* protective efficacy. a.i. concentration reflects reported formulation percentage and does not represent standardized airborne exposure or emanation rate; results should be interpreted accordingly

Further subgroup analyses indicated that PE values varied by deployment setting (indoors *versus* outdoors) and transfluthrin a.i. concentration categories (Fig. 4). The > 20% w/v concentration category showed a nonsignificant pooled PE in this subgroup analysis (on the basis of three studies), whereas most (103/112 datasets) reports had tested ≤ 10% w/v concentrations (Fig. 4). A significant interaction was apparent between transfluthrin a.i. concentration and VPSR deployment setting (*β* = 1.92, *P* = 0.031), suggesting that lower concentrations (≤ 10% w/v) may be more effective when deployed outdoors compared with indoors, and vice versa when using higher concentrations (> 10% w/v) (Fig. S2a, b). VPSR diffuser type also influenced VPSR efficacy, with passive diffusers appearing more effective than coils, although the latter finding was based on ten datasets from two studies (Table S3). PE values were not dissimilar between mosquito genera (Figs. 4 and S5).

#### Effect of metofluthrin on mosquito numbers according to trap type

**Fig. 5:**
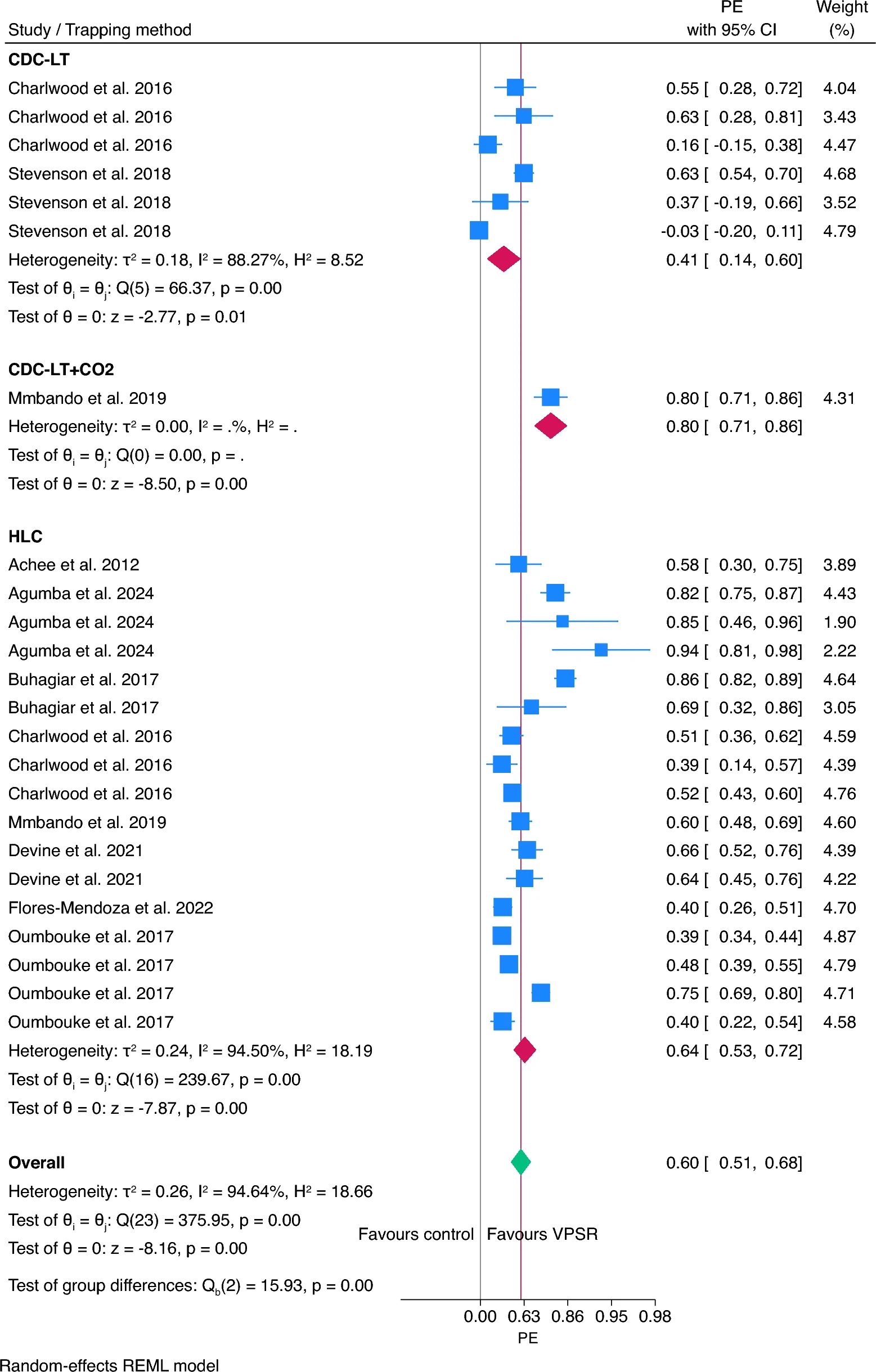
Forest plot showing the efficacy of metofluthrin on mosquito numbers measured by different trapping methods. Studies appearing more than once indicate multiple independent datasets reported within the same study. The size of the blue squares (mean of the estimates) reflects the sample size. *HLC* human landing catch, *CDC-LT* Centers for Disease Control light trap; *CDC-LT + CO₂* Centers for Disease Control carbon dioxide–baited light trap, *VPSR* volatile pyrethroid spatial repellent, *PE* protective efficacy

Mosquitoes were enumerated by HLC (8 studies, 17 datasets), CDC-LT (2 studies, 6 datasets), and CDC-LT + CO_2_ (1 study, 1 dataset). The respective datasets indicated a significant protective effect (*P* < 0.001 in each case), though PE values varied significantly (*Qβ*(2) = 15.93, *P* < 0.001) (Fig. 5).

Differences in PE values between trap types was examined by meta-regression and post-estimation pairwise comparisons: relative to CDC-LTs, values derived from both CDC-LT + CO_2_ (*P* = 0.051) and HLC (*P* = 0.053) methods tended to be higher. Trap type explained a modest proportion of between-study variability in PE estimates (*r*^2^ = 16.1%).

Subgroup analyses of the metofluthrin datasets (Fig. S7) suggested that indoor versus outdoor setting, a.i concentration category, and mosquito genera did not strongly influence PE values (*P* ≥ 0.09 in each case), whereas VPSR diffuser type did (*Qβ*(2) = 12.43, *P* < 0.001) (Fig. S7).

#### PE estimates generated from mosquito numbers using concurrent trapping methods

A total of 6 studies (27 datasets) provided mosquito numbers generated by concurrent HLC, CDC-LT, and CDC-LT + CO_2_ trapping, in the presence of transfluthrin (*n* = 4 studies) or metofluthrin (*n* = 2 studies) VPSRs. PE values for transfluthrin and metofluthrin VPSRs were not dissimilar (Fig. S6a,b), whereas trapping method explained ~21% (*r*^2^ = 20.7%) of the between-study variance (Fig. S17). Estimates generated from CDC-LT + CO_2_ data (88%, 95% CI 20–98%) were significantly higher than those from either HLC (62%, 95% CI 55–67%) (*β* = −1.14, *P* = 0.008) or CDC-LT (55%, 95% CI 42–66%) (*β* = −1.28, *P* = 0.004) data; values of the latter two categories were not dissimilar (*β* = −0.15, *P* = 0.65) (Figs. S6 and S17).

VPSRs were effective across mosquito genera *Anopheles*, *Culex*, and *Mansonia*, with marginal differences in PE values between them (*Qβ*(2) = 5.83, *P* = 0.05) (Fig. S5).

### Leishmaniasis and Phlebotomus sand flies

#### Effect of VPSRs on sand-fly-borne human infection incidence

A single study evaluated the effect of transfluthrin on *Leishmania* infection incidence with a value PE = 48% (95% CI 26–63%, *P* < 0.001), which is not dissimilar to the protective estimates against *Plasmodium* or ABV infections above [36].

#### Effect of VPSRs on sand fly numbers according to trapping method

Five studies reported VPSR impacts on sand fly numbers, but employed different insecticide compounds (transfluthrin, metofluthrin, cis–trans allethrin, and prallethrin), suggesting cis–trans allethrin was the most effective (Fig. S1). Two additional trapping methods (single studies using manual aspiration or suction traps) were reported. Pooled trapping methods indicated a significant overall PE of 81% (95% CI 63–91%, *P* < 0.001), though their respective pooled values differed significantly (*Qβ*(4) = 22.61, *P* < 0.001); due to the very small number of studies contributing to each trapping method, pairwise post-estimation comparisons were not performed.

**Fig. 6:**
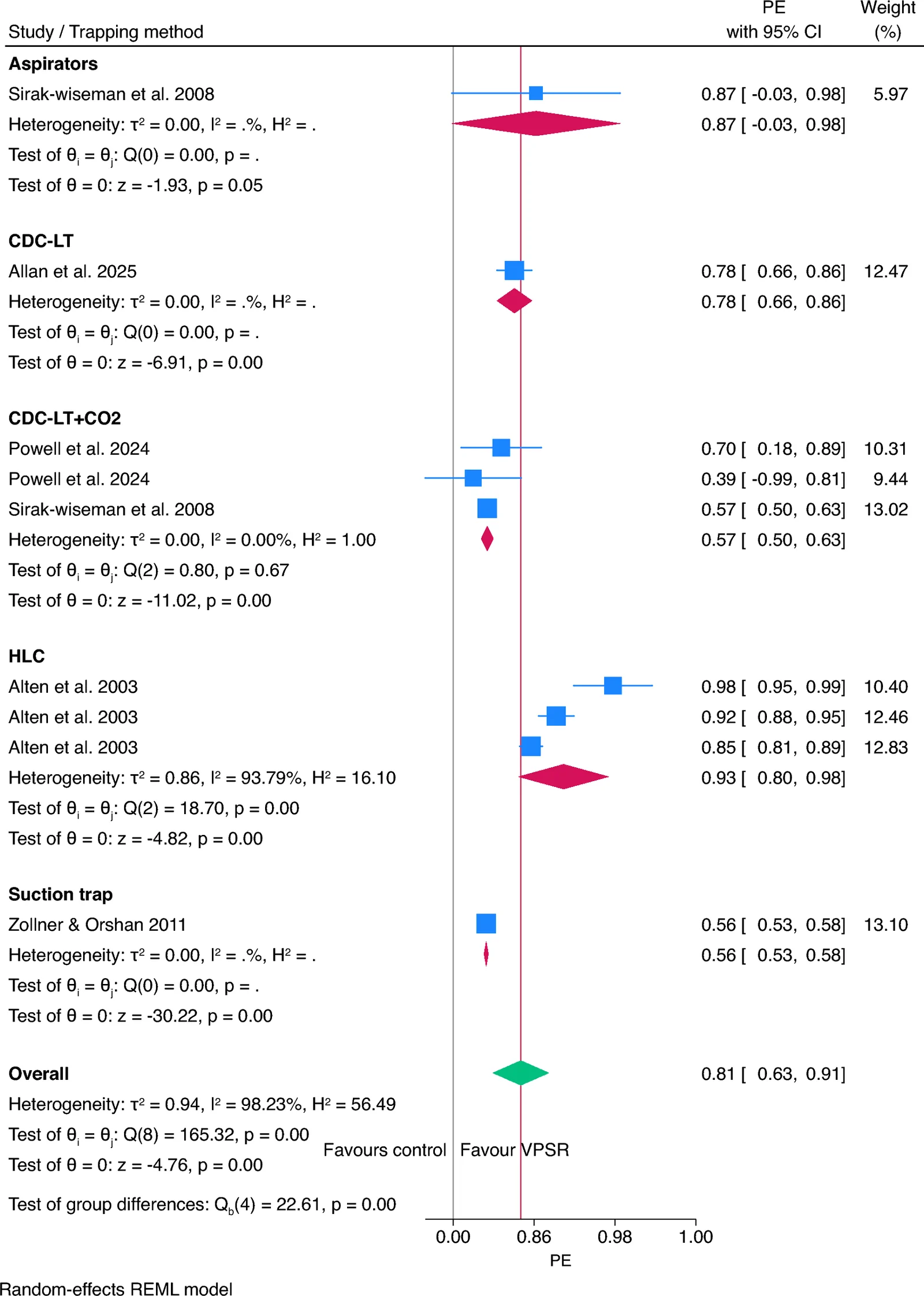
Forest plot showing the effect of VPSR on sand fly numbers estimated using different trapping methods. Studies appearing more than once indicate multiple independent datasets reported within the same study. The size of the blue squares (mean of the estimate) reflects the sample size. *HLC* human landing catch, *CDC-LT* Center for Disease Control light trap, *CDC-LT + CO*_*2*_ Center for Disease Control carbon dioxide-baited light trap, *VPSR* volatile pyrethroid spatial repellent, *PE* protective efficacy

Further subgroup analyses showed no differences associated with indoor (PE = 78%, 95% CI 67–86%) and outdoor (PE = 81%, 95% CI 55–92%) VPSR setting (*P* > 0.78) (Fig. S8), whereas there was a dependence on VPSR diffuser type (*P* < 0.01): data using Thermacell emanators provided the largest PE value (Fig. S8), as was observed for mosquitoes (Figs. 6 and S7).

### PE estimates generated from vector numbers by concurrent VPSR deployment in indoor versus outdoor settings

**Fig. 7:**
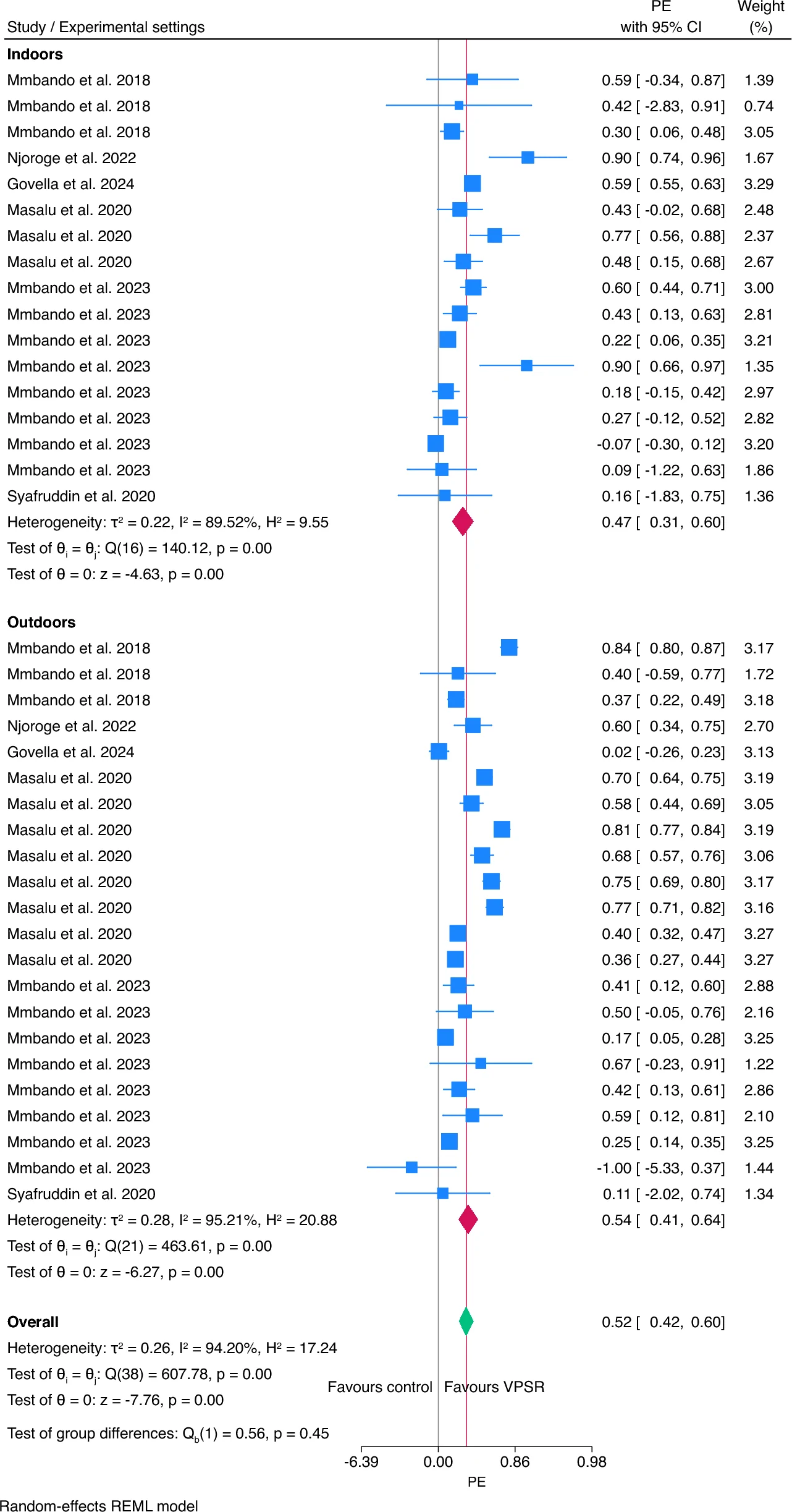
Forest plot showing the protective effect of transfluthrin against mosquito numbers in different deployment settings (indoors versus outdoors). Studies appearing more than once indicate multiple independent datasets reported within the same study. The size of the blue squares (mean of the estimate) reflects the sample size. *VPSR* volatile pyrethroid spatial repellent, *PE* protective efficacy

Ten studies concurrently measured the efficacy of VPSRs against mosquito numbers when deployed in indoor and outdoor settings: transfluthrin (*n* = 6 studies) and metofluthrin (*n* = 3 studies), and one study using prallethrin against sand flies [77]. The pooled estimates indoors (PE = 55%, 95% CI 41–65%, *P* < 0.001) versus outdoors (PE = 56%, 95% CI 45–64%, *P* < 0.001) were not dissimilar (Fig. S3), confirmed from analysis of transfluthrin and metofluthrin VPSR data separately (Figs. 7 and S4, respectively).

The single concurrent comparison of VPSR deployment settings against sand flies used prallethrin, with marginal significant effect indoors (PE = 87%, 95% CI −0.03 to 0.98), but high significant effect outdoors PE = 0.57 (95% CI 0.50–0.63, *P* < 0.001) [77].

### Small-study effects and publication bias

Significant heterogeneity was observed in the meta-analyses of transfluthrin (*I*^*2*^ = 98.2%) and of metofluthrin (*I*^*2*^ = 94.6%) on mosquito numbers (Figs. 3 and 5), and VPSRs combined effects on sand fly numbers (*I*^*2*^ = 97.9%) (Fig. 6). The respective subgroup analyses suggested that most measured variables contributed to inter-category heterogeneity, whereas VPSR deployment setting and mosquito genera tended not to (Figs. 4, S7, and S8).

Egger’s test provided significant evidence of small-study effects or publication bias for metofluthrin (*Z* = −2.43, *P* = 0.015) and transfluthrin (*Z* = −3.42, *P* < 0.001) on mosquito numbers (Figs. S9 and S10), but not for VPSRs combined effects on sand fly numbers (Z = −0.42, *P* = 0.673) (Fig. S12). Nonparametric trim-and-fill analyses to address publication bias did not identify any imputed studies, suggesting that coefficient adjustments would not improve model fits (Figs. S13 and S14). Sensitivity analyses for the effects of transfluthrin and metofluthrin on mosquito numbers did not identify any individual studies that notably contributed to the observed heterogeneity (Figs. S15 and S16).

## Discussion

This review extends prior VPSR syntheses by incorporating epidemiological endpoints alongside entomological outcomes, updating the literature to August 2025, and including available evidence on sand flies and leishmaniasis. The review identified an absence of extensive VPSR studies on human infection and substantial heterogeneity in entomological outcomes. Therefore, the generalizability and interpretation of the findings requires some caution.

Meta-analysis of the three large cRCTs suggest that transfluthrin VPSRs reduced *Plasmodium* infection incidence by PE = 50% (95% CI 21–69%) (Fig. 2), and ABV (dengue and Zika) incidence by a similar amount PE = 34% (95% CI 10–52%), the latter based on a single study [10]. Evidence for transfluthrin efficacy against *Leishmania* infection incidence was similarly limited, with one study reporting PE = 48% (95% CI 26–63%) [36]. Current evidence suggests that metofluthrin is unlikely to be effective against *Plasmodium* (PE = 35%, 95% CI −3.75 to 91%) [12], although this conclusion also remains uncertain because of the small number of available studies.

### Inference from entomological studies

Due to the general lack of human infection studies, VPSR efficacy is often inferred from the attributed changes in vector numbers, hence one purpose of this study was to identify how comparable PE estimates are when generated from epidemiological compared with entomological data. It should be stressed, however, that estimates from the latter data are not equivalents, nor replace the need to gather estimates from epidemiological data.

Vector numbers are measured using different entomological trapping methods, the most common being HLC, CDC-LT, and CDC-LTs + CO_2_. For the purposes of the current analysis, the latter category included CDC-LTs coupled with CO_2_ dispensers (*n* = 2), and CDC-LTs co-located in human occupied sleeping quarters (*n* = 4). Most entomological VPSR studies were conducted on mosquitoes (only five studies on sand flies), demonstrating significant reductions according to the data from each trap type.

Significant variation between trap types was detected by the six studies (four of transfluthrin, two of metofluthrin) that deployed all trap types concurrently, whereby CDC-LTs + CO_2_ produced significantly higher values than those generated using either CDC-LTs alone (*P* < 0.001) or HLCs (*P* < 0.001), the latter two being not dissimilar. These results suggest a probable dependence on trapping method.

PE values were also generated from the nonconcurrent studies of transfluthrin; while subgroup estimates indicated borderline differences between trap types, meta-regression showed that these differences were not statistically significant. Conversely, metofluthrin studies suggested probable differences, with higher values equally from HLCs and CDC-LTs + CO_2_ compared with CDC-LTs alone, although trap type explained only a modest proportion of between-study variability.

Across the entomological meta-analyses, heterogeneity was high (*I*^2^ > 90%), indicating substantial variability in effect sizes across studies. Accordingly, pooled PE estimates should be interpreted as average effects across diverse contexts and experimental designs rather than as a single generalizable effect applicable to all settings. This heterogeneity likely reflects the differences in combined study-specific design features, variably attributed to trapping method, vector taxa, deployment setting, intervention design, active ingredient concentration, and other unidentified features. Subgroup analyses help to describe how estimates differed across study features, but did not eliminate residual heterogeneity within categories.

One limitation of entomologically derived PE values is that the strength of evidence to infer a potential epidemiological impact against infection is not equal across trap types: HLCs quantify blood-seeking female mosquitoes and sand flies attracted to host odor cues and body temperature [79, 80] alighting to blood-feed, resulting in a potential infectious bite. In contrast, CDC-LTs, in absence of odor cues, attract phototaxic insects using light as a predominant visual cue [79, 81], consequently trapping both male and female individuals and in different physiological and gonotrophic states.

As the more proximate measure to the human biting rate, HLC data are often treated as the gold standard for measuring the PEs of VPSRs from entomological data [9, 59]. Therefore, it was expected that estimates derived from human infection incidence data might be more consistently aligned to estimates calculated from HLC and CDC-LT + CO_2_ data rather than from CDC-LT data. Scaling is unreliable as absolute counts of female vectors using CDC-LTs and HLCs rarely correlate, and transfluthrin PE values calculated from concurrent infection data and vector counts do not correlate either [10, 13, 36]. While reductions in vector density derived from these trapping methods provide valuable entomological insights, they remain indirect measures of transmission risk. Higher-order indices, such as sporozoite rates and entomological inoculation rates (EIR), offer stronger linkage to human infection risk, but were reported by only two of the five VPSR human infection studies included in this review [12, 13] (Fig. 2). Incorporation of such measures into entomological studies would improve our understanding of the relationships between entomological and epidemiological data.

### Indoor versus outdoor settings

The recent recommendation of transfluthrin diffusers by the WHO [3] was conditional on their deployment indoors. Consequently, this meta-analysis also focused on comparing VPSR deployment settings. For the purposes of analysis, indoor and outdoor categories were defined on the basis of the published descriptions; the former studies conducted within fully or semi-enclosed physical structures, the latter not restricted by physical structures. All the human infection VPSR studies were conducted indoors.

Considering the entomological studies, pooling the data from all trapping methods, transfluthrin and methofluthrin VPSRs each were effective in reducing mosquito numbers when deployed in indoor (PE = 45–62%) and outdoor (PE = 52–62%) settings (Figs. 4 and S7). Similarly, estimates calculated from studies that provided data from paired indoor/outdoor experiments did not reveal significant differences (indoors: PE = 55%, 95% CI 41–65%; outdoors: PE = 56%, 95% CI 45–64%) (Fig. S3).

Against sand flies, 7/9 datasets were VPSR studies conducted outdoors, not least due to the general exophilic/exophagic behavior of Phlebotomus sand fly vectors (Fig. S8). Pooling compounds suggested high efficacy outdoors (PE = 81%, 95% CLs 55%, 92%) and not dissimilar to values from indoor settings (Fig. S8). Paired data from a single study reported that prallethrin VPSR was highly effective in reducing numbers of *P. papatasi* sand fly vectors in tents but only marginally significant in bedrooms [77].

The collective results of the entomological studies suggest that the potential protection against human-vector contact provided by outdoor deployment of VPSRs is not likely to be inferior to that provided by indoor deployment. Notwithstanding, there was a high degree of variation in deployment settings and experimental designs that inevitably may have resulted in some misclassification.

### Repellent a.i. concentrations

Only a single dose–response study was identified by this review. Conducted in outdoor settings in Thailand, the study reported a positive linear association between the level of repellency of *An. minimus* and increasing transfluthrin a.i. concentration over a range of 1.5–20% w/v [75]. Performing a linear regression on the reported data, we estimate that for every 5% increase in a.i. concentration, there was a 10% increase in repellency (*r*^2^ = 0.802); no clear asymptote in the degree of repellency was detected by 20% w/v. Trends in the lethality effects were similar.

Analysis of the non-dose–response studies of transfluthrin or methofluthrin provided inconsistent evidence of a dose–response effect on mosquito repellency, however, the majority of transfluthrin datasets (93/112) tested a.i. concentrations of ≤ 5% w/v (Fig. 4). As these subgroup analysis findings are not derived from a direct within-study dose–response study, the results should be treated with caution; more dose–response studies are needed. Another important caveat in comparing a.i. concentrations between pyrethroid compounds is that each compound has a distinct chemical structure, level of volatility, and potency. Metofluthrin has a higher volatility and is more potent, hence requires lower a.i. concentrations compared with transfluthrin. Allethrin and prallethrin are of low-to-moderate relative volatility and potency as spatial repellents. Hence, pooling the few results from the diversity of compounds tested on sand flies, for example, should be treated with caution. Specific compound dose–response studies are needed by which to also confirm the apparent a.i. concentration × deployment setting interaction.

### Vector taxa

Subgroup analyses of transfluthrin or metafluthrin VPSRs did not reveal statistical difference in PE values derived from mosquito genera-specific data (Figs. 4 and S7), in contrast to the concurrent study datasets that revealed modest variations between genera (*Qβ*(2) = 5.83, *P* = 0.05); highest PE values were indicated from data on *Anopheles* (70%, 95% CI 52–81%), followed by *Mansonia* (68%, 95% CI 66–69%) and *Culex* (52%, 95% CI 35–65%) (Fig. S5). Inter-genera or population behavioral and/or physiological traits likely influence the magnitude of VPSR repellency. For example, responses to transfluthrin-treated eave ribbons and sandals (≤ 5% w/v) in Tanzania showed significant reductions in numbers of *An. arabiensis* (PE = 53%, 95% CI 0.34–0.65) and *Culex* spp. (PE = 27%, 95% CI 0.60–0.88), but not *An. funestus* or *Mansonia* spp. [47]. Also in Tanzania, transfluthrin-treated sisal ribbons (2% w/v) placed in kitchen doorways significantly reduced the entry of *Culex* spp. (PE = 77%, 95% CI 56–87) but not *An. arabiensis* [46].

### Considerations for VPSR deployment

The potential advantages of VPSRs over traditional vector control methods include their portability for operational deployment and potential cost savings depending on the delivery device, effective duration, and spatial range of the VPSR volatile.

VPSR delivery device types vary in design, practicality, and cost. Passive diffusers (emanators) range from simple impregnated hessian, plastic strips, or ribbons placed to encircle users [50, 68], to more structured portable devices such as self-standing plastic units or small sachets attached to clothing or furniture [11, 55, 61]. Passive diffusers are simple to use, require no power source, and employ inexpensive, widely available materials such as hessian or jute, as they are durable fabrics [68]. Hessian and polyethylene-based systems can be maintained at minimal cost, making them highly scalable, and emerge as the most cost-effective current delivery device type [36, 43, 44, 46, 47, 52, 54, 66, 68–71, 75, 76]. Of the other VPSR systems, thermacell-type emanators maintain stable a.i. release rates through battery-powered heat dispersion [17, 62, 65, 77], but are both more costly and battery dependent. The volatility of the VPSR compound helps to determine which delivery devices are more suitable.

The duration of effectiveness from a single deployment will determine the inter-intervention interval, and number of repeat interventions per unit time necessary to maintain coverage; short durations are less potentially feasible. Manufacturer recommendations and real-life studies indicate effective durations of 1–12 months, rarely > 12 months [50, 57]. The SC Johnson Guardian™ spatial repellent is claimed to persist for up to 12 months [57], whereas hessian fabric transfluthrin emanators (2% w/v a.i.) located in outdoor settings last for up to 3–6 months [43, 68], and treated ribbons and coils require replacement within 1 month [39] and 24 h [12, 15, 16], respectively. Against Phlebotomus sand flies, resin transfluthrin diffuser impregnated with 1200 mg a.i. and releasing at 5 mg per 24 h lasts for 35 days [76]. The PE of transfluthrin passive emanators against *Ae. aegypti* has been shown to decline over time, showing significant protection at 3 months (PE = 46.4%, 95% CI 41.1–51.8%), but not at 6 months (PE = 2.2%, 95% CI −9.0 to 14.0%) [58].

The spatial range of the VPSR will in part determine the number of units required per user/household. Manufacturers of transfluthrin-based products generally recommend placing them 1–3 m distance from users [82, 83]. A single study assessed the gradient of protection by placing 0.03% w/v transfluthrin coils at increasing distances from 0.3 to 30 m from the human (“point source”) under semi-field experimental conditions [15]. At 0.3 m of distance, the proportion of *An. gambiae* that fed on volunteers was 20% with a single coil (“point source”) versus 65% with no coil, and 4% with coils on both sides (“bubble”) versus 86% with no coil; effectiveness decreased with distance, with no significant effect at 30 m. Similar observations of *Ph. papatasi* sand fly numbers at 2.3 m, 4.6 m, and 7.6 m from the VPSR (cis–trans allethrin, a.i. concentration > 20% w/w), gave PE values of 97.7%, 92.5%, and 87.5%, respectively [62]. These data suggest that for every 1-m increase in distance from the host, the mean PE value decreases by ~2% (*r*^2^ = 0.992).

Studies of vector resistance to VPSR chemicals are generally lacking. The single study included in this review reported that transfluthrin (≤ 5% w/v) applied to window eaves was effective against Tanzanian *Anopheles* strains highly resistant to pyrethroids [74]. Neither was resistance to transfluthrin detected when wild pyrethroid-resistant *An. arabiensis* in Tanzania and *An. gambiae* s.l. in Benin were exposed to the WHO-prequalified Mosquito Shield™ spatial emanator [42, 55]. By contrast, *An. gambiae* s.s. in Kenya exhibited reduced behavioral sensitivity to transfluthrin, with elevated transcription levels of the cytochrome P450 gene CYP12F12, and high frequencies of knockdown resistance (kdr) mutations L995F and L995S [84]. This highlights the necessity for continual surveillance of resistant markers.

## Conclusions

The meta-analyses indicate that the tested VPSR products can reduce mosquito and sand fly numbers captured in traps when deployed indoors or outdoors. There is also limited evidence of protection against the incidence of mosquito-borne infections and *Leishmania* infection from a small number of trials. Thus, VPSRs should be considered a useful complementary vector control method, but key evidence gaps remain—particularly data on epidemiological outcomes. Efficacy estimates derived from entomological data may be strongly influenced by trapping method, but their absolute values are unlikely to correlate with values obtained through infection/clinical studies, and therefore best used comparatively. Ultimately, counts of vector numbers do not account for vector population demographic structure, biting behavior, physiological status, vectorial capacity, and other characteristics that can strongly influence individual transmission risk and infection spread [85, 86]. In addition to the challenging work to validate entomological markers of protection, information on optimal a.i. concentrations, their effective persistence, and cost-effectiveness are critically needed for programmatic and sustainable scale-up.

## Supplementary Information


Additional file 1.

## Data Availability

All data generated or analyzed during this study are included in this published article and its supplementary information files.

## Abbreviations

ABV: *Aedes*-borne viral infections (dengue or Zika)
a.i.: Active ingredient
BIREME: Latin American and Caribbean Center on Health Sciences Information
CDC: Centers for Disease Control and Prevention
CDC-LT: CDC light trap
CDC-LT + CO₂: CDC light trap baited/enhanced with carbon dioxide (or co-located with human odor cues, as defined in methods)
CI: Confidence interval
CLIMOS: Climate Monitoring and Decision Support Framework for Sand Fly-borne Diseases Detection and Mitigation
CO₂: Carbon dioxide
COV: Coefficient of variation
cRCT: Cluster randomized controlled trial
CRD: Controlled release device
EIR: Entomological inoculation rate
HLC: Human landing catch
HLR: Human landing rate
HR: Hazard ratio
ICC: Intraclass correlation coefficient
ICTRP: WHO International Clinical Trials Registry Platform
IRR: Incidence rate ratio
IRS: Indoor residual spraying
ITN/ITNs: Insecticide-treated net(s)
LLIN/LLINs: Long-lasting insecticide-treated net(s)
LILACS: Latin American and Caribbean Health Sciences Literature database
MEDLINE: Medical Literature Analysis and Retrieval System Online
NIH: National Institutes of Health
nRCT: Nonrandomized controlled trial
OR: Odds ratio
Ovid: Ovid (database/search platform used for MEDLINE/Embase)
PCR: Polymerase chain reaction
PE: Protective efficacy
PRISMA: Preferred Reporting Items for Systematic Reviews and Meta-Analyses
PROSPERO: International Prospective Register of Systematic Reviews
RCT: Randomized controlled trial
REMs: Random-effects models
RR: Rate ratio/risk ratio (as used in meta-analysis methods)
SE: Standard error
STATA: Stata statistical software
ULV: Ultra-low-volume (spatial spraying)
VPSR/VPSRs: Volatile pyrethroid spatial repellent(s)
w/v: Weight per volume
w/w: Weight per weight
WHO: World Health Organization
