# Supplementary material for: Efficacy of volatile pyrethroid spatial repellents to reduce exposure to mosquito and sand fly vectors and human infection risk: a systematic review and meta-analysis
Source: Parasit Vectors. 2026 Jun 18;19:370. doi: 10.1186/s13071-026-07393-2 (PMC13523321; doi:10.1186/s13071-026-07393-2)
Supplement: Supplementary file 1 [file 13071_2026_7393_MOESM1_ESM.docx]

**Supporting information (S)**

**Table of content:**

| 1. Search strategies | 2 |
| --- | --- |
| 2. Characteristics and quality assessment of included studies | 5 |
| Table S1: PRISMA 2020 checklist | 5 |
| Table S2: Quality assessment using National Institutes of Health Quality Assessment Tools | 8 |
| Table S3: Characteristics of included studies | 10 |
| 3. Forest plots | 12 |
| Fig.S1. Forest plot showing the effect of VPSR on sand fly numbers | 12 |
| Fig.S2. Forest plot showing the effect of transfluthrin a.i. concentration indoor vs outdoor   a. a.i. concentration <10% W/V b. a.i. concentration >10% W/V | 13  13  14 |
| Fig.S3. Forest plot showing the protective effect (PE) of VPSR against mosquito numbers, generated concurrently in indoors vs. outdoors settings. | 15 |
| Fig.S4. Forest plot showing the PE of metofluthrin against mosquito numbers, generated concurrently in indoors vs. outdoors settings. | 16 |
| Fig.S5. Forest plot showing the PE of VPSRs against mosquito numbers, by genus. | 17 |
| Fig.S6. Forest plot showing the PE of VSPRs against mosquito numbers generated concurrently using different trapping methods in the same study. | 18 |
| Fig.S6a. Forest plot showing the PE of transfluthrin against mosquito numbers generated concurrently using different trapping methods in the same study. | 19 |
| Fig.S6b. Forest plot showing the PE of metofluthrin against mosquito numbers generated concurrently using different trapping methods in the same study. | 20 |
| Fig.S7. Forest plot showing subgroup analysis of the effect of metofluthrin on mosquito numbers (PE) across study characteristics. | 21 |
| Fig.S8 Forest plot showing subgroup analysis of the combined effect of VPSR compounds on sand fly numbers (PE) across study characteristics | 22 |
| 4. Funnel plots | 23 |
| Fig.S9. Funnel plot for publication bias – metofluthrin on mosquito numbers | 23 |
| Fig.S10. Funnel plot for publication bias – transfluthrin on mosquito numbers | 23 |
| Fig.S11. Funnel plot for publication bias – transfluthrin on infection incidence | 24 |
| Fig.S12. Funnel plot for publication bias – on sand fly numbers. | 24 |
| 5. Nonparametric trim-and-fill tests | 25 |
| Fig.S13. Nonparametric trim-and-fill tests for metofluthrin on mosquito numbers | 25 |
| Fig.S14. Nonparametric trim-and-fill tests for transfluthrin on mosquito numbers | 25 |
| 6. Sensitivity analyses | 26 |
| Fig.S15. Sensitivity analyses for metofluthrin on mosquito numbers | 26 |
| Fig.S16. Sensitivity analyses for transfluthrin on mosquito numbers | 27 |
| 7. PROSPERO protocol registration | 28 |
| 8. Meta regression   Fig.S17. Meta-regression comparing HLCs and CDC-LTs | 29  29 |
| Fig.S18. Meta-regression testing interaction between transfluthrin concentration and deployment setting (indoor vs outdoor) | 29 |

1. **Search strategies**

**Ovid MEDLINE(R) ALL <1946 to March 19, 2025>**

#1	pyrethroid.mp.	(7367)

#2	spatial repellent.mp.	(148)

#3	1 and 2	(54)

#4	("spatial repellent" or "volatile pyrethroid" or "metofluthrin" or "transfluthrin" or "prallethrin" or "allethrin").mp. [mp=title, book title, abstract, original title, name of substance word, subject heading word, floating sub-heading word, keyword heading word, organism supplementary concept word, protocol supplementary concept word, rare disease supplementary concept word, unique identifier, synonyms, population supplementary concept word, anatomy supplementary concept word]	(587)

#5	pyrethroid spatial repellents.mp.	(7)

#6	("mosquito*" or "sand fly" or "sand fl*").mp. [mp=title, book title, abstract, original title, name of substance word, subject heading word, floating sub-heading word, keyword heading word, organism supplementary concept word, protocol supplementary concept word, rare disease supplementary concept word, unique identifier, synonyms, population supplementary concept word, anatomy supplementary concept word]	(68028)

#7	4 and 6	(337)

#8	((("passive diffuser" or "vector control" or "hessian fabric, vaporizer mat*,") and "emanator") or "hessian" or "coil" or "thermacell").mp. [mp=title, book title, abstract, original title, name of substance word, subject heading word, floating sub-heading word, keyword heading word, organism supplementary concept word, protocol supplementary concept word, rare disease supplementary concept word, unique identifier, synonyms, population supplementary concept word, anatomy supplementary concept word]	(55020) (55926)

#9	4 and 8	(67)

*Screened for duplicates: #3 + #7 + #9 = 458*

**Embase Classic+Embase <1947 to 2025 Week 11>**

#1	pyrethroid/	(11070)

#2	spatial repellent.mp.	(206)

#3	1 and 2	(52)

#4	("spatial repellent" or "volatile pyrethroid" or "metofluthrin" or "transfluthrin" or "prallethrin" or "allethrin").mp. [mp=title, abstract, heading word, drug trade name, original title, device manufacturer, drug manufacturer, device trade name, keyword heading word, floating subheading word, candidate term word]	(1018)

#5	pyrethroid spatial repellents.mp.	(9)

#6	("mosquito*" or "sand fly" or "sand fl*").mp. [mp=title, abstract, heading word, drug trade name, original title, device manufacturer, drug manufacturer, device trade name, keyword heading word, floating subheading word, candidate term word]	(84085)

#7	4 and 6	(467)

#8	((("passive diffuser" or "vector control" or "hessian fabric, vaporizer mat*,") and "emanator") or "hessian" or "coil" or "thermacell").mp. [mp=title, abstract, heading word, drug trade name, original title, device manufacturer, drug manufacturer, device trade name, keyword heading word, floating subheading word, candidate term word]	(91574)

#9	4 and 8	(90)

*Screened for duplicates: #3 + #7 + #9 = 609*

**Web of Science**

#1	pyrethroid.mp.	(13146)

#2	spatial repellent.mp.	(697)

#3	1 and 2	(86)

#4	("spatial repellent" or "volatile pyrethroid" or "metofluthrin" or "transfluthrin" or "prallethrin" or "allethrin").mp. [mp=title, book title, abstract, original title, name of substance word, subject heading word, floating sub-heading word, keyword heading word, organism supplementary concept word, protocol supplementary concept word, rare disease supplementary concept word, unique identifier, synonyms, population supplementary concept word, anatomy supplementary concept word]	(807)

#5	pyrethroid spatial repellents.mp.	(86)

#6	("mosquito*" or "sand fly" or "sand fl*").mp. [mp=title, book title, abstract, original title, name of substance word, subject heading word, floating sub-heading word, keyword heading word, organism supplementary concept word, protocol supplementary concept word, rare disease supplementary concept word, unique identifier, synonyms, population supplementary concept word, anatomy supplementary concept word]	(81933)

#7	4 and 6	(383)

#8	((("passive diffuser" or "vector control" or "hessian fabric, vaporizer mat*,") and "emanator") or "hessian" or "coil" or "thermacell").mp. [mp=title, book title, abstract, original title, name of substance word, subject heading word, floating sub-heading word, keyword heading word, organism supplementary concept word, protocol supplementary concept word, rare disease supplementary concept word, unique identifier, synonyms, population supplementary concept word, anatomy supplementary concept word]	(150773)

#9	4 and 8	(87)

*Screened for duplicates: #3 + #7 + #9 = 556*

**LILACS (BIREME)**

#1	pyrethroid.mp.	(316)

#2	spatial repellent.mp.	(27)

#3	1 and 2	(6)

#4	("spatial repellent" or "volatile pyrethroid" or "metofluthrin" or "transfluthrin" or "prallethrin" or "allethrin").mp. [mp=title, book title, abstract, original title, name of substance word, subject heading word, floating sub-heading word, keyword heading word, organism supplementary concept word, protocol supplementary concept word, rare disease supplementary concept word, unique identifier, synonyms, population supplementary concept word, anatomy supplementary concept word]	(34)

#5	pyrethroid spatial repellents.mp.	(4)

#6	("mosquito*" or "sand fly" or "sand fl*").mp. [mp=title, book title, abstract, original title, name of substance word, subject heading word, floating sub-heading word, keyword heading word, organism supplementary concept word, protocol supplementary concept word, rare disease supplementary concept word, unique identifier, synonyms, population supplementary concept word, anatomy supplementary concept word]	(15527)

#7	4 and 6	(25)

#8	((("passive diffuser" or "vector control" or "hessian fabric, vaporizer mat*,") and "emanator") or "hessian" or "coil" or "thermacell").mp. [mp=title, book title, abstract, original title, name of substance word, subject heading word, floating sub-heading word, keyword heading word, organism supplementary concept word, protocol supplementary concept word, rare disease supplementary concept word, unique identifier, synonyms, population supplementary concept word, anatomy supplementary concept word]	(1208)

#9	4 and 8	(5)

*Screened for duplicates: #3 + #7 + #9 = 36*

2. **Characteristics and quality assessment of included studies**

**Table S1. PRISMA 2020 Checklist**

| **Section and Topic** | **Item #** | **Checklist item** | **Location where item is reported** |
| --- | --- | --- | --- |
| **TITLE** | | |  |
| Title | 1 | Identify the report as a systematic review. | 1 |
| **ABSTRACT** | | |  |
| Abstract | 2 | See the PRISMA 2020 for Abstracts checklist. | 2 |
| **INTRODUCTION** | | |  |
| Rationale | 3 | Describe the rationale for the review in the context of existing knowledge. | 4-6 |
| Objectives | 4 | Provide an explicit statement of the objective(s) or question(s) the review addresses. | 6 |
| **METHODS** | | |  |
| Eligibility criteria | 5 | Specify the inclusion and exclusion criteria for the review and how studies were grouped for the syntheses. | 6 |
| Information sources | 6 | Specify all databases, registers, websites, organisations, reference lists and other sources searched or consulted to identify studies. Specify the date when each source was last searched or consulted. | 7-8 |
| Search strategy | 7 | Present the full search strategies for all databases, registers and websites, including any filters and limits used. | Additional file 1 |
| Selection process | 8 | Specify the methods used to decide whether a study met the inclusion criteria of the review, including how many reviewers screened each record and each report retrieved, whether they worked independently, and if applicable, details of automation tools used in the process. | 8 |
| Data collection process | 9 | Specify the methods used to collect data from reports, including how many reviewers collected data from each report, whether they worked independently, any processes for obtaining or confirming data from study investigators, and if applicable, details of automation tools used in the process. | 8, Additional file 1 |
| Data items | 10a | List and define all outcomes for which data were sought. Specify whether all results that were compatible with each outcome domain in each study were sought (e.g. for all measures, time points, analyses), and if not, the methods used to decide which results to collect. | Additional file 1 |
|  | 10b | List and define all other variables for which data were sought (e.g. participant and intervention characteristics, funding sources). Describe any assumptions made about any missing or unclear information. | Additional file 1 |
| Study risk of bias assessment | 11 | Specify the methods used to assess risk of bias in the included studies, including details of the tool(s) used, how many reviewers assessed each study and whether they worked independently, and if applicable, details of automation tools used in the process. | 10, Additional file 1 |
| Effect measures | 12 | Specify for each outcome the effect measure(s) (e.g. risk ratio, mean difference) used in the synthesis or presentation of results. | 9 |
| Synthesis methods | 13a | Describe the processes used to decide which studies were eligible for each synthesis (e.g. tabulating the study intervention characteristics and comparing against the planned groups for each synthesis (item #5)). | Additional file 1 |
|  | 13b | Describe any methods required to prepare the data for presentation or synthesis, such as handling of missing summary statistics, or data conversions. | 9-10 |
|  | 13c | Describe any methods used to tabulate or visually display results of individual studies and syntheses. | Additional file 1 |
|  | 13d | Describe any methods used to synthesize results and provide a rationale for the choice(s). If meta-analysis was performed, describe the model(s), method(s) to identify the presence and extent of statistical heterogeneity, and software package(s) used. | 9-10  Additional file 1 |
|  | 13e | Describe any methods used to explore possible causes of heterogeneity among study results (e.g. subgroup analysis, meta-regression). | 9-10  Additional file 1 |
|  | 13f | Describe any sensitivity analyses conducted to assess robustness of the synthesized results. | 10  Additional file 1 |
| Reporting bias assessment | 14 | Describe any methods used to assess risk of bias due to missing results in a synthesis (arising from reporting biases). | 10  Additional file 1 |
| Certainty assessment | 15 | Describe any methods used to assess certainty (or confidence) in the body of evidence for an outcome. | 10  Additional file 1 |
| **RESULTS** | | |  |
| Study selection | 16a | Describe the results of the search and selection process, from the number of records identified in the search to the number of studies included in the review, ideally using a flow diagram. | 11 |
|  | 16b | Cite studies that might appear to meet the inclusion criteria, but which were excluded, and explain why they were excluded. | 12 |
| Study characteristics | 17 | Cite each included study and present its characteristics. | Additional file 1 |
| Risk of bias in studies | 18 | Present assessments of risk of bias for each included study. | Additional file 1 |
| Results of individual studies | 19 | For all outcomes, present, for each study: (a) summary statistics for each group (where appropriate) and (b) an effect estimate and its precision (e.g. confidence/credible interval), ideally using structured tables or plots. | 13-26 |
| Results of syntheses | 20a | For each synthesis, briefly summarise the characteristics and risk of bias among contributing studies. | Additional file 1 |
|  | 20b | Present results of all statistical syntheses conducted. If meta-analysis was done, present for each the summary estimate and its precision (e.g. confidence/credible interval) and measures of statistical heterogeneity. If comparing groups, describe the direction of the effect. | 13-26  Additional file 1 |
|  | 20c | Present results of all investigations of possible causes of heterogeneity among study results. | 26 |
|  | 20d | Present results of all sensitivity analyses conducted to assess the robustness of the synthesized results. | 26 |
| Reporting biases | 21 | Present assessments of risk of bias due to missing results (arising from reporting biases) for each synthesis assessed. | Additional file 1 |
| Certainty of evidence | 22 | Present assessments of certainty (or confidence) in the body of evidence for each outcome assessed. | 26  Additional file 1 |
| **DISCUSSION** | | |  |
| Discussion | 23a | Provide a general interpretation of the results in the context of other evidence. | 27-32 |
|  | 23b | Discuss any limitations of the evidence included in the review. | 27-32 |
|  | 23c | Discuss any limitations of the review processes used. | 27-32 |
|  | 23d | Discuss implications of the results for practice, policy, and future research. | 27-33 |
| **OTHER INFORMATION** | | |  |
| Registration and protocol | 24a | Provide registration information for the review, including register name and registration number, or state that the review was not registered. | 6  Additional file 1 |
|  | 24b | Indicate where the review protocol can be accessed, or state that a protocol was not prepared. | 6 |
|  | 24c | Describe and explain any amendments to information provided at registration or in the protocol. | None |
| Support | 25 | Describe sources of financial or non-financial support for the review, and the role of the funders or sponsors in the review. | 34 |
| Competing interests | 26 | Declare any competing interests of review authors. | 35 |
| Availability of data, code and other materials | 27 | Report which of the following are publicly available and where they can be found: template data collection forms; data extracted from included studies; data used for all analyses; analytic code; any other materials used in the review. | Additional file 1 |

**Table S2. Quality assessment of included studies using National Institutes of Health Quality Assessment of Controlled Intervention Studies.**

| **Study** | **Study design** | **National Institutes of Health Quality Assessment Tools question number and score allocated ^a^** | | | | | | | | | | | | | | | | |
| --- | --- | --- | --- | --- | --- | --- | --- | --- | --- | --- | --- | --- | --- | --- | --- | --- | --- | --- |
|  |  | **1** | **2** | **3** | **4** | **5** | **6** | **7** | **8** | **9** | **10** | **11** | **12** | **13** | **14** | **Rating** | **Raters initials** |  |
| Achee et al. [16] | nRCT | N | NA | Y | NA | NR | Y | NA | NA | Y | Y | Y | N | Y | NA | Fair | NR & OC |  |
| Agumba et al. [65] | nRCT | N | NA | Y | NA | NR | Y | NA | NA | Y | Y | Y | N | Y | NA | Fair | NR & OC |  |
| Ahebwa et al. [66] | nRCT | N | NA | Y | NA | NR | Y | NA | NA | Y | Y | Y | N | Y | NA | Fair | NR & OC |  |
| Allan et al. [36] | cRCT | Y | Y | Y | NA | NR | Y | NA | NA | Y | Y | Y | N | Y | Y | Good | NR & OC |  |
| Alten et al. [62] | RCT | Y | Y | Y | NA | NR | Y | NA | NA | Y | Y | Y | N | Y | Y | Good | NR & MB |  |
| Andrés et al. [39] | RCT | Y | Y | Y | NA | NR | Y | NA | NA | Y | Y | Y | N | Y | Y | Good | NR & OC |  |
| Buhagiar et al. [64] | nRCT | N | NA | Y | NA | NR | Y | NA | NA | Y | Y | Y | N | Y | NA | Fair | NR & OC |  |
| Burton et al. [37] | nRCT | N | NA | Y | NA | NR | Y | NA | NA | Y | Y | Y | N | Y | NA | Fair | NR & OC |  |
| Charlwood et al. [67] | nRCT | N | NA | Y | NA | NR | Y | NA | NA | Y | Y | Y | N | Y | NA | Fair | NR & MB |  |
| Devine et al. [40] | RCT | Y | Y | Y | NA | NR | Y | NA | NA | Y | Y | Y | N | Y | Y | Good | NR & OC |  |
| Flores-Mendoza et al. [41] | RCT | Y | Y | Y | NA | NR | Y | NA | NA | Y | Y | Y | N | Y | Y | Good | NR & MB |  |
| Fongnikin et al. [42 | RCT | Y | Y | Y | NA | NR | Y | NA | NA | Y | Y | Y | Y | Y | Y | Good | NR & OC |  |
| Govella et al. [68] | nRCT | N | NA | Y | NA | NR | Y | NA | NA | Y | Y | Y | N | Y | NA | Fair | NR & OC |  |
| Govella et al. [43] | RCT | Y | Y | Y | NA | NR | Y | NA | NA | Y | Y | Y | N | Y | Y | Good | NR & OC |  |
| Hill et al. [14] | RCT | Y | Y | Y | Y | Y | Y | Y | Y | Y | Y | Y | N | Y | Y | Good | NR & OC |  |
| Maia et al. [38] | RCT | Y | Y | Y | Y | N | Y | NA | NA | Y | Y | Y | N | Y | Y | Good | NR & OC |  |
| Masalu et al. [44] | RCT | Y | Y | Y | NA | NR | Y | NA | NA | Y | Y | Y | N | Y | Y | Good | NR & OC |  |
| Masalu et al. [45] | RCT | Y | Y | Y | NA | NR | Y | NA | NA | Y | Y | Y | N | Y | Y | Good | NR & OC |  |
| Masalu et al. [46] | RCT | Y | Y | Y | NA | NR | Y | NA | NA | Y | Y | Y | N | Y | Y | Good | NR & OC |  |
| Mmbando et al. [69] | nRCT | N | NA | Y | NA | NR | Y | NA | NA | Y | Y | Y | N | Y | NA | Fair | NR & OC |  |
| Mmbando et al. [70] | nRCT | N | NA | Y | NA | NR | Y | NA | NA | Y | Y | Y | N | Y | NA | Fair | NR & MB |  |
| Mmbando et al. [47] | RCT | Y | Y | Y | NA | NR | Y | NA | NA | Y | Y | Y | Y | Y | Y | Good | NR & OC |  |
| Moreno-Gómez et al. [48] | RCT | Y | Y | Y | NA | NR | Y | NA | NA | Y | Y | Y | N | Y | Y | Good | NR & MB |  |
| Morrison et al. [10] | cRCT | Y | Y | Y | Y | Y | Y | Y | Y | Y | Y | Y | Y | Y | Y | Good | NR & OC |  |
| Njoroge et al. [49] | RCT | Y | Y | Y | NA | NR | Y | NA | NA | Y | Y | Y | N | Y | Y | Good | NR & MB |  |
| Njoroge et al. [71] | nRCT | N | NA | Y | NR | Y | Y | NA | NA | Y | Y | Y | N | Y | NA | Fair | NR & OC |  |
| Ochomo et al. [11] | cRCT | Y | Y | Y | NR | NR | Y | NA | NA | Y | Y | Y | Y | Y | Y | Good | NR & OC |  |
| Ogoma et al. [15] | RCT | Y | Y | Y | NA | NR | Y | NA | NA | Y | Y | Y | N | Y | Y | Good | NR & OC |  |
| Ogoma et al. [50] | RCT | Y | Y | Y | Y | Y | Y | NA | NA | Y | Y | Y | N | Y | Y | Good | NR & OC |  |
| Oumbouke et al. [51] | RCT | Y | Y | Y | NA | NR | Y | NA | NA | Y | Y | Y | N | Y | Y | Good | NR & OC |  |
| Powell et al. [76] | nRCT | N | NA | Y | NA | NR | Y | NA | NA | Y | Y | Y | N | Y | NA | Fair | NR & MB |  |
| Sangoro et al. [52] | RCT | Y | Y | Y | Y | NR | Y | NA | NA | Y | Y | Y | N | Y | Y | Good | NR & OC |  |
| Sirak-wiseman et al. [77] | nRCT | N | NA | Y | NA | NR | Y | NA | NA | Y | Y | Y | N | Y | NA | Fair | NR & OC |  |
| Stevenson et al. [53 | RCT | Y | Y | Y | NA | NR | Y | NA | NA | Y | Y | Y | N | Y | Y | Good | NR & OC |  |
| Sukkanon et al. [72] | nRCT | N | NA | Y | NR | Y | Y | NA | NA | Y | Y | Y | N | Y | NA | Fair | NR & OC |  |
| Supreme et al. [54] | RCT | Y | Y | Y | NA | NR | Y | NA | NA | Y | Y | Y | N | Y | Y | Good | NR & OC |  |
| Swai et al. [56 | RCT | Y | Y | Y | NA | NR | Y | NA | NA | Y | Y | Y | N | Y | Y | Good | NR & MB |  |
| Swai et al. [55] | RCT | Y | Y | Y | NA | NR | Y | NA | NA | Y | Y | Y | N | Y | Y | Good | NR & OC |  |
| Swai et al. [57] | RCT | Y | Y | Y | NA | NR | Y | NA | NA | Y | Y | Y | N | Y | Y | Good | NR & MB |  |
| Syafruddin et al. [12] | cRCT | Y | Y | Y | Y | Y | Y | Y | Y | Y | Y | Y | N | Y | Y | Good | NR & OC |  |
| Syafruddin et al. [13] | cRCT | Y | Y | Y | Y | Y | Y | Y | Y | Y | Y | Y | Y | Y | Y | Good | NR & MB |  |
| Tambwe et al. [58] | RCT | Y | Y | Y | NA | NR | Y | NA | NA | Y | Y | Y | N | Y | Y | Good | NR & OC |  |
| Tambwe et al. [74] | nRCT | N | NA | Y | NA | NR | Y | NA | NA | Y | Y | Y | N | Y | NA | Fair | NR & MB |  |
| Tambwe et al. [73] | nRCT | N | NA | Y | NA | NR | Y | NA | NA | Y | Y | Y | N | Y | NA | Fair | NR & OC |  |
| Tambwe et al. [59] | RCT | Y | Y | Y | NA | NR | Y | NA | NA | Y | Y | Y | N | Y | Y | Good | NR & OC |  |
| Vajda et al. [60] | RCT | Y | Y | Y | NA | NR | Y | NA | NA | Y | Y | Y | N | Y | Y | Good | NR & OC |  |
| Vajda et al. [61] | RCT | Y | Y | Y | NA | NR | Y | NA | NA | Y | Y | Y | N | Y | Y | Good | NR & OC |  |
| Yan et al. [75] | nRCT | N | NA | Y | NA | NR | Y | NA | NA | Y | Y | Y | N | Y | NA | Fair | NR & MB |  |
| Zollner & Orshan [78] | nRCT | N | NA | Y | NA | NR | Y | NA | NA | Y | Y | Y | N | Y | NA | Fair | NR & OC |  |
| **Total** | |  |  |  |  |  |  |  |  |  |  |  |  |  |  | **Fair n=15 (33.3%)**  **Good n=30 (66.7%)** |  |  |

**Abbreviations:** Y – yes, N – no, NA – not applicable, NR – not repoprted, ^a^ The National Institutes of Health (NIH) Quality Assessment Tools (<https://www.nhlbi.nih.gov/health-topics/study-quality-assessment-tools>) were utilised for evaluating the quality of the included studies.The rating for assessing study quality is as follows: A study was classified as "good" if the potential bias was minimal, categorised as "fair" if there was a moderate risk of bias that didn't render the results unreliable, and labelled as "poor" if there was a substantial risk of bias that could considerably impact the interpretation of the results. Reviewer’s initials relate to manuscript authors: NR – Niba Rawlings, OC – Orin Courtenay, MB – Mark Bailey.

Assessed domains included selection bias (randomisation methods for RCTs and cRCTs, allocation concealment), performance bias (adherence to interventions for infection incidence studies), detection/measurement bias (blinding of households or areas and outcome assessors, and outcome assessment procedures), confounding/baseline imbalance (similarity of baseline characteristics between groups), and analytical bias/power bias (whether the study reported sufficient statistical power, ≥80%, to detect meaningful differences; this was rated based on information reported, rather than formally calculated, and contributed to the overall quality assessment.

**Table S3. Characteristics of included studies**

| **Study author & year** | **Experimental design** | **Study duration** | **Location** | **VPSR** | **Outcome measures** | **Vector** | **Collection method** | **VPSR diffuser type** | **Setting** | **Type of material** | **VPSRs a.i. concentration (w/v)*** |
| --- | --- | --- | --- | --- | --- | --- | --- | --- | --- | --- | --- |
| Achee et al. [16] | nRCT | 4 weeks | Thailand | Metofluthrin | Reduction in mosquito numbers | Mosquitoes | HLC | Coils | Indoors & Outdoors | Coil | ≤ 5% |
| Agumba et al. [65] | nRCT | 12 weeks | Kenya | Metofluthrin | Reduction in mosquito numbers | Mosquitoes | HLC | Thermacell emanators | Indoors & Outdoors | Device | NR |
| Ahebwa et al. [66] | nRCT | 6 weeks | Thailand | Transfluthrin | Reduction in mosquito numbers | Mosquitoes | HLC | Passive diffusers | Outdoors | Hessian & Cotton fabric | > 20% |
| Allan et al. [36] | cRCT | 56 weeks | Syria | Transfluthrin | Reduction in sand fly densities and CL incidence | Sand flies | CDC-LT | Passive diffusers | Indoors | Polyethylene | ≤ 5% |
| Alten et al. [62] | RCT | 1 week | Turkey | Cis-trans allethrin | Reduction in sand fly numbers | Sand flies | HLC | Thermacell emanators | Outdoors | Device | > 20% |
| Andrés et al. [39] | RCT | 4 weeks | Tanzania | Transfluthrin | Reduction in mosquito numbers | Mosquitoes | HLC | Passive diffusers | Outdoors | Polyethylene | ≤ 5% |
| Buhagiar et al. [64] | nRCT | 1 week | Australia | Metofluthrin | Reduction in mosquito numbers | Mosquitoes | HLC | Passive diffusers | Indoors | Polyethylene | > 5 - ≤10% |
| Burton et al. [37] | nRCT | 20 weeks | Zambia | Transfluthrin | Reduction in mosquito numbers | Mosquitoes | HLC | Passive diffusers | Outdoors | Polyethylene | NR |
| Charlwood et al. [67] | nRCT | 42 weeks | Cambodia | Metofluthrin | Reduction in mosquito numbers | Mosquitoes | HLC  CDC-LT | Passive diffusers | Outdoors | Polyethylene | > 5 - ≤10% |
| Devine et al. [40] | RCT | 24 weeks | Mexico | Metofluthrin | Reduction in mosquito numbers | Mosquitoes | HLC | Passive diffusers | Indoors | methacrylate polymer net | > 5 - ≤10% |
| Flores-Mendoza et al. [41] | RCT | 2 weeks | Peru | Metofluthrin | Reduction in mosquito numbers | Mosquitoes | HLC | Passive diffusers | Outdoors | Biodegradable | > 20% |
| Fongnikin et al. [42 | RCT | 5 weeks | Benin | Transfluthrin | Reduction in mosquito numbers | Mosquitoes | HLC | Passive diffusers | Indoors | Polyethylene | ≤ 5% |
| Govella et al. [68] | nRCT | 11 weeks | Tanzania | Transfluthrin | Reduction in mosquito numbers | Mosquitoes | HLC | Passive diffusers | Outdoors | Hessian fabric | ≤ 5% |
| Govella et al. [43] | RCT | 7 weeks | Tanzania | Transfluthrin | Reduction in mosquito numbers | Mosquitoes | HLC | Passive diffusers | Indoors & Outdoors | Hessian fabric | ≤ 5% |
| Hill et al. [14] | RCT | 24 weeks | China | Transfluthrin | Reduction in *Plasmodium* infection incidence | Mosquitoes | CDC-LT | Coils | Indoors | Coil | ≤ 5% |
| Maia et al. [38] | RCT | 24 weeks | Tanzania | Transfluthrin | Reduction in mosquito numbers | Mosquitoes | HLC | Coils | Indoors | Coil | ≤ 5% |
| Masalu et al. [44] | RCT | 12 weeks | Tanzania | Transfluthrin | Reduction in mosquito numbers | Mosquitoes | HLC | Passive diffusers | Outdoors | Sisal & Hessian fabric | ≤ 5% |
| Masalu et al. [45] | RCT | 4 weeks | Tanzania | Transfluthrin | Reduction in mosquito numbers | Mosquitoes | HLC | Passive diffusers | Outdoors | Sisal fabric | ≤ 5% |
| Masalu et al. [46] | RCT | 16 weeks | Tanzania | Transfluthrin | Reduction in mosquito numbers | Mosquitoes | HLC  CDC-LT+CO_2_ | Passive diffusers | Outdoors | Hessian fabric | ≤ 5% |
| Mmbando et al. [69] | nRCT | 11 weeks | Tanzania | Transfluthrin | Reduction in mosquito numbers | Mosquitoes | HLC  CDC-LT+CO_2_ | Passive diffusers | Indoors | Hessian fabric | ≤ 5% |
| Mmbando et al. [70] | nRCT | 36 weeks | Zimbabwe | Metofluthrin | Reduction in mosquito numbers | Mosquitoes | HLC  CDC-LT+CO_2_ | Passive diffusers | Outdoors | Hessian fabric | ≤ 5% |
| Mmbando et al. [47] | RCT | 28 weeks | Tanzania | Transfluthrin | Reduction in mosquito numbers | Mosquitoes | HLC  CDC-LT | Passive diffusers | Indoors & Outdoors | Hessian fabric | ≤ 5% & > 5 - ≤10% |
| Moreno-Gómez et al. [48] | RCT |  | Italy | Transfluthrin | Reduction in mosquito numbers | Mosquitoes | HLC | Spray | Outdoors | aerosol spray | ≤ 5% |
| Morrison et al. [10] | cRCT | 96 weeks | Peru | Transfluthrin | Reduction in Aedes-borne viral infection incidence and mosquito numbers | Mosquitoes | HLC | Passive diffusers | Indoors | Ployethylene | ≤ 5% |
| Njoroge et al. [49] | RCT | 3 weeks | Kenya | Transfluthrin | Reduction in mosquito numbers | Mosquitoes | HLC | Passive diffusers | Outdoors | Hessian fabric | ≤ 5% |
| Njoroge et al. [71] | nRCT | 28 weeks | Kenya | Transfluthrin | Reduction in mosquito numbers | Mosquitoes | HLC | Passive diffusers | Indoors & Outdoors | Hessian fabric | > 10 - ≤20% |
| Ochomo et al. [11] | cRCT | 120 weeks | Kenya | Transfluthrin | Reduction in *Plasmodium* infection incidence | Mosquitoes | HLC  CDC-LT | Passive diffusers | Indoors | NR | ≤ 5% |
| Ogoma et al. [15] | RCT | 1 week | Tanzania | Transfluthrin | Reduction in mosquito numbers | Mosquitoes | HLC | Coils | Outdoors | Coil | ≤ 5% |
| Ogoma et al. [50] | RCT | 120 weeks | Tanzania | Transfluthrin | Reduction in mosquito numbers | Mosquitoes | HLC | Passive diffusers | Outdoors | Hessian fabric | > 10 - ≤20% |
| Oumbouke et al. [51] | RCT | 20 weeks | Benin | Metofluthrin | Reduction in mosquito numbers | Mosquitoes | HLC | Coils | Indoors | Coil | ≤ 5% |
| Powell et al. [76] | nRCT | 5 weeks | Greece | Transfluthrin | Reduction in sand fly numbers | Sand flies | CDC-LT+CO_2_ | Passive diffusers | Outdoors | Polyethylene | > 10 - ≤20% |
| Sangoro et al. [52] | RCT | 76 weeks | Tanzania | Transfluthrin | Reduction in mosquito numbers | Mosquitoes | HLC | Passive diffusers | Indoors & Outdoors | Hessian fabric | ≤ 5% & > 5 - ≤10% |
| Sirak-wiseman et al. [77] | RCT | 20 weeks | Zambia | Metofluthrin | Reduction in mosquito numbers | Mosquitoes | CDC-LT | Passive diffusers | Indoors & Outdoors | Device | > 20% |
| Stevenson et al. [53 | nRCT | 10 weeks | Israel | Prallethrin | Reduction in sand fly numbers | Sand flies | CDC-LT+CO_2_, Aspirator | Thermacell emanators | Indoors & Outdoors | Device | ≤ 5% |
| Sukkanon et al. [72] | nRCT | 4 weeks | Thailand | Transfluthrin | Reduction in mosquito numbers | Mosquitoes | HLC | Passive diffusers | Indoors & Outdoors | Polyethylene | ≤ 5% |
| Supreme et al. [54] | RCT | 24 weeks | Haiti | Transfluthrin | Reduction in mosquito numbers | Mosquitoes | HLC | Passive diffusers | Indoors & Outdoors | Hessian fabric | ≤ 5% |
| Swai et al. [56 | RCT | 3 weeks | Tanzania | Transfluthrin | Reduction in mosquito numbers | Mosquitoes | HLC  CDC-LT+CO_2_ | Passive diffusers | Indoors | Polyethylene | ≤ 5% |
| Swai et al. [55] | RCT | 5 weeks | Tanzania | Transfluthrin | Reduction in mosquito numbers | Mosquitoes | HLC | Passive diffusers | Indoors | Polyethylene | ≤ 5% |
| Swai et al. [57] | RCT | 48 weeks | Tanzania | Transfluthrin | Reduction in mosquito numbers | Mosquitoes | HLC | Passive diffusers | Indoors | Polyethylene | ≤ 5% |
| Syafruddin et al. [12]^1^ | cRCT | 24 weeks | Indonesia | Metofluthrin | Reduction in *Plasmodium* infection incidence | Mosquitoes | HLC | Coils | Indoors | Coil | ≤ 5% |
| Syafruddin et al. [13]^1^ | cRCT | 96 weeks | Indonesia | Transfluthrin | Reduction in *Plasmodium* infection incidence | Mosquitoes | HLC | Passive diffusers | Indoors & Outdoors | NR | ≤ 5% |
| Tambwe et al. [58] | RCT | 48 weeks | Tanzania | Transfluthrin | Reduction in mosquito numbers | Mosquitoes | HLC | Passive diffusers | Outdoors | Hessian fabric | ≤ 5% |
| Tambwe et al. [74] | nRCT | 24 weeks | Tanzania | Transfluthrin | Reduction in mosquito numbers | Mosquitoes | HLC | Passive diffusers | Outdoors | Hessian fabric | ≤ 5% |
| Tambwe et al. [73] | nRCT | 40 weeks | Tanzania | Transfluthrin | Reduction in mosquito numbers | Mosquitoes | HLC | Passive diffusers | Outdoors | Hessian fabric | ≤ 5% |
| Tambwe et al. [59] | RCT | 7 weeks | Tanzania | Transfluthrin | Reduction in mosquito numbers | Mosquitoes | HLC | Passive diffusers | Indoors | Hessian fabric | ≤ 5% |
| Vajda et al. [60] | RCT | 4 weeks | Thailand | Transfluthrin | Reduction in mosquito numbers | Mosquitoes | HLC | Passive diffusers | Outdoors | paper | ≤ 5% |
| Vajda et al. [61] | RCT | 12 weeks | Cambodia | Transfluthrin | Reduction in mosquito numbers | Mosquitoes | HLC | Passive diffusers | Outdoors | NR | > 20% |
| Yan et al. [75] | nRCT | 3 weeks | Thailand | Transfluthrin | Reduction in mosquito numbers | Mosquitoes | HLC | Passive diffusers | Outdoors | Hessian & Cotton fabric | > 10 - ≤20% |
| Zollner & Orshan [78] | nRCT | 1 week | Israel | Metofluthrin | Reduction in sand fly numbers | Sand flies | Suction trap | Fan vapouriser | Outdoors | Device | > 20% |

**Abbreviation:** RCT – randomised control trial; cRCT – cluster randomised control trial; nRCT – non-randomised control trial; VPSR – volatile pyrethroid spatial repellent; a.i. – active ingredient; NR – Not reported.

***** VPSRs a.i. concentration from included studies were grouped into one of the following categories: ≤ 5%w/v, > 5 - ≤10%w/v, > 10 - ≤20% w/v and > 20% w/v.

**Note:** Passive diffusers are also called passive emanators.

**^1^** Only two Plasmodium studies reported sporozoite rates and EIR; incidence outcomes were rarely reported.

3. **Forest plots**

**Fig. S1. Forest plot showing the effect of VPSR on sand fly numbers.** Studies appearing more than once indicate multiple independent datasets reported within the same study. Transfluthrin (3 datasets, 2 studies), metofluthrin (1 dataset, 1 study), cis-trans allethrin (3 datasets, 1 study) and prallethrin (2 datasets, 1 study).

**Fig. S2. Forest plot showing the effect of transfluthrin a.i. concentration in indoor vs outdoor settings.** Studies appearing more than once indicate multiple independent datasets reported within the same study.

a. **a.i. concentration ≤ 10% W/V**

b. **a.i. concentration >10% W/V.** Studies appearing more than once indicate multiple independent datasets reported within the same study.

**Fig. S3. Forest plot showing the protective effect of VPSRs against mosquito numbers, generated concurrently in indoors vs. outdoors settings.** Studies appearing more than once indicate multiple independent datasets reported within the same study. The size of the blue squares (mean of the estimate) reflects the sample size. PE protective efficacy; VPSR volatile pyrethroid spatial repellent.

**Fig. S4.** **Forest plot showing the protective effect of metofluthrin against mosquito numbers, generated concurrently in indoors vs. outdoors settings.** Studies appearing more than once indicate multiple independent datasets reported within the same study. The size of the blue squares (mean of the estimate) reflects the sample size. PE protective efficacy; VPSR volatile pyrethroid spatial repellent.

**Fig. S5.** **Forest plot showing the protective effect of VPSRs against mosquito numbers, by genus.** Studies appearing more than once indicate multiple independent datasets reported within the same study. The size of the blue squares (mean of the estimate) reflects the sample size. PE protective efficacy; VPSR volatile pyrethroid spatial repellent.

**Fig. S6. Forest plot showing the protective effect of VSPRs against mosquito numbers generated concurrently using different trapping methods in the same study.** Studies appearing more than once indicate multiple independent datasets reported within the same study. The size of the blue squares (mean of the estimate) reflects the sample size. PE protective efficacy.

**Fig. S6a. Forest plot showing the protective effect of transfluthrin against mosquito numbers generated concurrently using different trapping methods in the same study.** Studies appearing more than once indicate multiple independent datasets reported within the same study. The size of the blue squares (mean of the estimate) reflects the sample size. PE protective efficacy.

**Fig. S6b. Forest plot showing the protective effect of metofluthrin against mosquito numbers generated concurrently using different trapping methods in the same study.** Studies appearing more than once indicate multiple independent datasets reported within the same study. The size of the blue squares (mean of the estimate) reflects the sample size. PE protective efficacy.

**Fig. S7. Forest plot showing subgroup analysis of the effect of metofluthrin on mosquito numbers (PE) across study characteristics.**

RCT randomised-controlled trials; nRCT non-randomised controlled trials; VPSR volatile pyrethroid spatial repellent; a.i. active ingredient (w/v); CDC-LT Center for Disease Control light trap; HLC human landing catches; CO₂ carbon dioxide.

**Fig. S8. Forest plot showing subgroup analysis of the combined effect of VPSR compounds on sand fly numbers (PE) across study characteristics**

RCT randomised-controlled trials; cRCT cluster randomised controlled trials; nRCT non-randomised controlled trials; PE protective efficacy, NR not reported; VPSR volatile pyrethroid spatial repellent; a.i. active ingredient (w/v); CDC-LT Center for Disease Control light trap; HLC human landing catches; CO₂ carbon dioxide.

4. **Funnel plots**

**Fig. S9. Funnel plot for publication bias – metofluthrin on mosquito numbers**

**Fig. S10. Funnel plot for publication bias – transfluthrin on mosquito numbers**

**Fig. S11. Funnel plot for publication bias – transfluthrin on infection incidence**

**Fig. S12. Funnel plot for publication bias – on sand fly numbers.**

5. **Nonparametric trim-and-fill tests**

**Fig. S13. Nonparametric trim-and-fill tests for metofluthrin on mosquito numbers**

**Fig. S14. Nonparametric trim-and-fill tests for transfluthrin on mosquito numbers**

6. **Sensitivity analysis**

**Fig. S15. Sensitivity analyses for metofluthrin on mosquito numbers**

**Fig. S16. Sensitivity analyses for transfluthrin on mosquito numbers**

7. **PROSPERO protocol registration**

8. **Meta-regression**

**Fig. S17. Meta-regression comparing HLCs and CDC-LTs**

**Fig. S18. Meta-regression testing interaction between transfluthrin concentration and deployment setting (indoor vs outdoor)**
